# Exploration of the longitudinal clinical course and potential prognostic factors in Parkinson’s disease: 2.5-year observational study in Japan

**DOI:** 10.3389/fneur.2026.1845594

**Published:** 2026-05-26

**Authors:** Kei-ichi Ishikawa, Yushin Tominaga, Yu Kigoshi-Tansho, Kentaro Ohta, Ayami Suzuki, Yuanzhe Li, Wado Akamatsu, Shinji Saiki, Nobutaka Hattori

**Affiliations:** 1 Department of Neurology, Faculty of Medicine, Juntendo University, Tokyo, Japan; 2 Center for Genomic and Regenerative Medicine, Graduate School of Medicine, Juntendo University, Tokyo, Japan; 3 Neuroscience Clinical Development, R&D, Johnson and Johnson Innovative Medicine, Tokyo, Japan; 4 Department of Neurology, Institute of Medicine, University of Tsukuba, Tsukuba, Japan; 5 Department of Diagnosis, Prevention and Treatment of Dementia, Graduate School of Medicine, Juntendo University, Tokyo, Japan; 6 Neurodegenerative Disorders Collaborative Laboratory, RIKEN Center for Brain Science, Saitama, Japan

**Keywords:** Parkinson’s disease, longitudinal observational study, clinically relevant worsening, orthostatic hypotension, Unified Parkinson’s Disease Rating Scale

## Abstract

**Background:**

Several long-term studies have characterized the course of Parkinson’s disease (PD) outside Japan, but few have been conducted in Japan. This study aimed to explore the longitudinal clinical course of PD symptoms in patients receiving standard therapy in Japan and to identify potential factors associated with clinically relevant worsening.

**Methods:**

This study used data from 348 patients with PD at Juntendo University Hospital, Japan. Baseline characteristics and longitudinal clinical status, including the Unified Parkinson’s Disease Rating Scale (UPDRS) motor scores, were obtained and summarized. Factors potentially associated with clinically relevant worsening were also explored.

**Results:**

Among 194 analyzed patients, the study population predominantly had mild to moderate PD, with a mean age of 66.4 years, mean disease duration of 6.22 years, mean UPDRS III score of 20.0, and mean Hoehn and Yahr stage of 2.2. Most patients were receiving L-dopa (88.7%), with a mean daily dose of 477.0 mg. The mean UPDRS III score improved by 3.3 points at the 6-month visit and remained relatively stable for up to 2.5 years. In multivariate exploratory analyses, baseline motor severity, time from PD onset to referral, and orthostatic hypotension were associated with clinically relevant worsening.

**Conclusion:**

Our findings suggest that timely referral to specialized care and assessment of autonomic symptoms, particularly orthostatic hypotension, may help identify patients at higher risk of clinically relevant worsening in routine PD care.

## 1 Introduction

Parkinson’s disease (PD) is a multisystem progressive neurodegenerative disorder caused by the preferential loss of dopaminergic neurons in the substantia nigra pars compacta and other brain regions (1). PD is characterized by motor symptoms, including bradykinesia, rigidity and tremor, and non-motor symptoms, including neuropsychiatric, autonomic, and sensory symptoms (2–5). It is estimated that approximately 10 million people worldwide and ~200,000 people in Japan are affected by PD (6, 7).

Multiple studies have reported the longitudinal course of the disease in large cohorts of non-Japanese (8–10) and Japanese patients (11, 12). To the best of our knowledge, no studies have characterized the longitudinal clinical course and explored factors associated with clinically relevant worsening using other disease severity indicators, such as the Unified Parkinson’s Disease Rating Scale (UPDRS), in Japanese patients with PD. This study aimed to explore the longitudinal clinical course of PD symptoms in patients receiving standard therapies in Japan and to examine potential factors associated with clinically relevant worsening of PD symptoms. Clinically relevant worsening was assessed using several prespecified exploratory criteria, including changes in motor impairment and treatment escalation, to evaluate practical indicators of short-term worsening.

## 2 Methods

### 2.1 Data source

The study protocol complied with the principles of the Declaration of Helsinki and was approved by the Ethics Committee of Juntendo University (approved no. 2012157). Patients who visited the hospital between September 2019 and June 2022 and provided written informed consent were enrolled. This study used a PD database containing information from 348 patients who were diagnosed with PD based on the 2015 MDS clinical diagnostic criteria for PD (3), received standard care at Juntendo University Hospital, Tokyo, Japan, and consented to the study. Although diagnosis was based on the MDS clinical diagnostic criteria, motor symptoms were assessed using the original UPDRS according to routine clinical practice at the study site. Clinical data were obtained from medical records from September 2019, corresponding to the first patient’s first visit, to September 2022, corresponding to the last patient’s last visit. In this study, motor symptoms were evaluated using the UPDRS III and IV and the HY staging scale at baseline and subsequent routine standard-of-care clinic visits every 6 months for up to 2.5 years. The assessments were conducted during the “ON” state. Demographic and clinical information was recorded at baseline and/or subsequent visits. These included sex, date of birth, weight and height, year of PD onset, treatment duration, histories of constipation, orthostatic hypotension (OH), rapid eye movement (REM) sleep behavior disorder (RBD), and hyposmia, all of which were determined by medical interview; cognition and depressive symptoms measured by the Mini-Mental State Examination (MMSE) and Hamilton Depression Rating Scale-6 (HAMD-6), and prescription data for PD treatment. To evaluate the longitudinal disease course of PD symptoms, only patients with an available UPDRS III total score at least 1.5 years after baseline were included in the analysis. The analysis excluded patients whose diagnoses changed to diseases other than PD during the study period and patients who were judged by the investigator to the ineligible for this study.

In the present study, PD-related genetic mutations were analyzed only in selected patients with rapid motor worsening and were not systematically assessed in the entire cohort. Details of the genetic mutation analyses are provided in the Supplementary material.

### 2.2 Statistical analysis

Analyses used SAS (version 9.4) and R (version 4.3.3). Continuous variables were summarized using descriptive statistics, including mean, standard deviation (SD), median, and range, while categorical variables were presented as frequencies and percentages. Mean values were used as reference values for selected cut-offs in subsequent exploratory analyses.

A Cox proportional hazards regression model was used and fitted using the Breslow method (13) to explore the candidate factors associated with clinically relevant worsening. For exploratory purposes, we prespecified several indicators of clinically relevant worsening and treatment escalation over follow-up, including ≥5-point worsening in the UPDRS III score, a ≥ 25% increase in the UPDRS III score, a ≥ 1-point increase in the HY stage, a 1.2- to 1.5-fold increase in L-dopa dosage, a 2- to 3-fold increase in L-dopa equivalent dose (LED), and a UPDRS IV total score reaching ≥2 points. These measures were used as pragmatic exploratory outcomes and not as validated definitions of disease progression. The model estimated hazard ratios (HRs) with 95% confidence intervals (CIs). Similarly, logistic regression model analyses were performed to explore factors associated with clinically relevant worsening at the 24-month time point and the model estimated odds ratios (ORs) with 95% CIs. These two models were applied as complementary approaches. The Cox model incorporated time-to-event information, whereas the 24-month landmark logistic model evaluated associations at a fixed, clinically interpretable time point. Concordant findings across both models were regarded as more robust, whereas model-specific findings were considered hypothesis-generating. The Wald test was used to evaluate the statistical significance of each coefficient in the model. Associations between selected factors and clinically relevant worsening were visually explored using Kaplan–Meier curves, when appropriate.

Univariate analysis was first conducted using these models to identify variables significantly associated with at least one predefined clinically relevant worsening event, adjusting for baseline PD medication use. In the univariate analysis, the following potential or known characteristics were included in each model: age, sex, age at onset, MMSE score, daily L-dopa dose, daily LED, and time from the onset of motor symptoms to referral to Juntendo University (categorized as equal to or greater than the mean vs. less than the mean), status of RBD, constipation, hyposmia, OH, type of motor impairment (akinetic-rigid/mixed/tremor-dominant), baseline disease severity (baseline UPDRS III total score), and family history of PD (classified as yes or no). Next, the identified variables were evaluated using a multivariate analysis. For comparisons of baseline characteristics, Student’s t-test was used for continuous variables, and Fisher’s exact test was used for categorical variables. All tests were 2-sided, with *p* values less than 0.05 considered statistically significant. Multiple-test corrections were not performed due to the exploratory nature of this study.

## 3 Results

Of the 348 patients with PD in the database, 194 were included in the analysis. Fourteen patients were excluded because their diagnosis changed during the evaluation period, one patient was excluded because of familial PD, and 139 were excluded because the evaluation period was less than 1.5 years. The mean (SD) ages at study entry and disease onset were 66.4 (10.01) and 60.9 (11.0) years, respectively. Most patients (85.1%) experienced disease onset at age 50 or older, while 14.9% were classified as early-onset PD as per the definition of the International Parkinson and Movement Disorder Society Task Force (14). The mean (SD) disease duration from motor symptom onset to baseline was 6.22 (5.25) years. The baseline demographic and clinical characteristics are summarized in Table 1.

**Table 1:** Baseline demographic and clinical characteristics.

Subject characteristics	Patients (*N* = 194)
Sex	
Male, *n* (%)	87 (44.8)
Female, *n* (%)	107 (55.2)
Height (cm)^a^	
Mean (SD)	160.4 (9.9)
Age at onset of PD	
<50, *n* (%)	29 (14.9)
≥50, *n* (%)	165 (85.1)
Mean (SD)	60.9 (11.0)
Age at entry	
Mean (SD)	66.4 (10.1)
Disease duration (year)	
Mean (SD)	6.22 (5.25)
<Mean, *n* (%)	122 (62.9)
≥Mean, *n* (%)	72 (37.1)
Family history of PD, *n* (%)	
First degree	7 (3.6)
Second degree	9 (4.6)
Third degree	9 (4.6)
Fourth degree	1 (0.5)
None	142 (73.2)
Unknown	26 (13.4)
Time from onset of motor symptoms to referral to Juntendo University Hospital (days)	
Mean (SD)	2359.4 (1874.7)
<Mean, *n* (%)	119 (61.3)
≥Mean, *n* (%)	75 (38.7)
Hoehn and Yahr Score^b^	
1 *n* (%)	47 (24.2)
2 *n* (%)	61 (31.4)
3 *n* (%)	55 (28.4)
4 *n* (%)	9 (4.6)
5 *n* (%)	3 (1.5)
<3, *n* (%)	108 (55.7)
≥3, *n* (%)	67 (34.5)
Mean (SD)	2.2 (1.0)
UPDRS III total score	
Mean (SD)	20.0 (12.6)
<Mean, *n* (%)	112 (57.7)
≥Mean, *n* (%)	82 (42.3)
UPDRS IV score	
0 *n* (%)	191 (98.5)
2 *n* (%)	2 (1.0)
4 *n* (%)	1 (0.5)
Current use of L-dopa	
Yes, *n* (%)	172 (88.7)
No, *n* (%)	22 (11.3)
Daily L-dopa dose (mg/day)^c^	
Mean (SD)	477.0 (237.7)
≤300, *n* (%)	67 (34.5)
>300, ≤600, *n* (%)	68 (35.1)
>600, ≤900, *n* (%)	30 (15.5)
>900, ≤1,200, *n* (%)	7 (3.6)
>1,200, *n* (%)	0
Unknown, *n* (%)	22 (11.3)
Daily L-dopa equivalent dose^d^	
Mean (SD)	616.6 (364.8)
≤Mean, *n* (%)	112 (57.7)
>Mean, *n* (%)	77 (39.7)
Unknown, *n* (%)	5 (2.6)

a *N* = 186.

b *N* = 174 excluding 19 with missing value.

c *N* = 172, excluding 22 with L-dopa dose = 0.

d *N* = 189 excluding 5 with missing value.

The mean values of the UPDRS III total scores and changes from baseline at each visit are summarized in Table 2. At baseline, the mean (SD) UPDRS III total score was 20.0 (12.6), ranging from 2.0 to 64, with a mean HY stage of 2.2 (1.0). The majority (88.7%) were receiving L-dopa with a mean dose (SD) of 477.0 (237.7) mg/day, and a mean LED of 616.6 (364.8) mg/day. Of the 194 patients, all but 3 (98.5%) had a UPDRS IV score of 0 at baseline.

**Table 2:** UPDRS-III scores and change from baseline at each visit.

VISIT	*N*	UPDRS III score	Change from baseline
Baseline	194	20.0 (12.6)	—
Month 6	186	16.8 (11.4)	−3.3 (8.2)
Month 12	184	17.3 (11.1)	−2.8 (8.6)
Month 18	186	17.0 (11.4)	−3.2 (8.9)
Month 24	163	17.5 (11.3)	−2.8 (9.7)
Month 30	124	17.3 (9.8)	−3.0 (12.9)

Mean (SD).

Changes in the UPDRS III score over time showed improvement at Month 6, with a mean (SD) change from baseline of −3.3 (8.2), indicating improvement in motor scores. The mean score remained relatively stable throughout the remaining observation period, up to Month 30. However, the large SD indicated high variability among patients, with some experiencing worsening in the UPDRS III score during this period. Of the 194 patients, 69 (35.6%) experienced worsening of five points or more in their UPDRS III score over the 2.5-year (30-month) observation period (Kaplan–Meier curve is presented as Supplementary Figure S1). The numbers of patients who met the predefined clinically relevant worsening criteria are summarized in Supplementary Table S1.

Supplementary material summarizes the exploration of rapid motor worsening on an individual patient basis. The baseline characteristics of 19 patients with rapid motor worsening, defined as the top 10% with the greatest annual worsening in UPDRS III score from baseline, are listed, and no specific common pattern in the baseline characteristics was observed within this subpopulation. Only one patient had a known pathogenic GBA mutation (see Supplementary Tables S17, S18 for detail). No significant differences in the patient characteristics were observed between patients with rapid motor worsening and the 57 patients who remained stable or improved (Supplementary Table S19).

To provide complementary perspectives on factors associated with clinically relevant worsening, we report results from both the Cox proportional hazards and the logistic regression models in parallel.

### 3.1 Cox proportional hazards model analysis

The results of the univariate analysis for clinically relevant worsening, defined as worsening of the UPDRS III score by five points or more from the baseline, are presented in Table 3. This analysis is presented first because it identified the largest number of significant factors (four) among the exploratory outcomes related to motor impairment or daily PD medication dose. Disease severity, as indicated by the baseline UPDRS III score, status of hyposmia, time from the onset of motor symptoms to referral to Juntendo University, and status of OH, were significantly associated with this outcome after adjustment for baseline PD medication use.

**Table 3:** Cox proportional hazards model (Univariate analysis): Time to worsening of UPDRS III total score defined as a 5-point increase from baseline.

Variable	Category	*N*	With event (%)	HR	95% CI	Wald test	Adjusted HR*	95% CI	Wald test
Total number of patients		194	69 (35.6%)						
Sex	Male	87	29 (33.3%)	Reference			Reference		
	Female	107	40 (37.4%)	1.14	0.71–1.84	*p* = 0.593	1.08	0.67–1.76	*p* = 0.745
Age	<Mean	87	26 (29.9%)	Reference			Reference		
	≥Mean	107	43 (40.2%)	1.35	0.83–2.20	*p* = 0.226	1.40	0.86–2.29	*p* = 0.176
Age at onset of PD	<50	29	9 (31.0%)	Reference			Reference		
	≥50	165	60 (36.4%)	1.13	0.56–2.27	*p* = 0.741	1.09	0.54–2.20	*p* = 0.813
Status of RBD	Yes	41	18 (43.9%)	1.50	0.88–2.57	*p* = 0.137	1.49	0.87–2.54	*p* = 0.150
	No	153	51 (33.3%)	Reference			Reference		
Daily L-dopa dose	≤Mean	105	32 (30.5%)	Reference			Reference		
	>Mean	89	37 (41.6%)	1.40	0.87–2.26	*p* = 0.162	1.51	0.92–2.45	*p* = 0.100
LED	≤Mean	112	33 (29.5%)	Reference			Reference		
	>Mean	77	31 (40.3%)	1.43	0.87–2.33	*p* = 0.157	–	–	–
Disease severity (baseline UPDRS III Score)	≤Mean	112	49 (43.8%)	Reference			Reference		
	>Mean	82	20 (24.4%)	0.49	0.29–0.82	*p* = 0.007	0.50	0.29–0.84	*p* = 0.009
Status of hyposmia	Yes	61	25 (41.0%)	2.12	1.10–4.09	*p* = 0.024	2.03	1.04–3.95	*p* = 0.037
	No	68	14 (20.6%)	Reference			Reference		
Time from the onset of motor symptoms to referral to a specialized hospital (Juntendo University)	<Mean	119	36 (30.3%)	Reference			Reference		
	≥Mean	75	33 (44.0%)	1.54	0.96–2.48	*p* = 0.072	1.64	1.01–2.66	*p* = 0.044
Status of orthostatic hypotension	Yes	44	22 (50.0%)	1.79	1.07–3.00	*p* = 0.027	1.74	1.03–2.93	*p* = 0.039
	No	140	42 (30.0%)	Reference			Reference		
Family history of Parkinson’s disease	First degree	7	3 (42.9%)	0.98	0.31–3.15	*p* = 0.976	1.01	0.31–3.24	*p* = 0.991
	Second degree	9	3 (33.3%)	0.81	0.25–2.60	*p* = 0.722	0.83	0.26–2.66	*p* = 0.754
	Third degree	9	2 (22.2%)	0.59	0.14–2.41	*p* = 0.460	0.60	0.15–2.48	*p* = 0.482
	Fourth degree	1	0 (0.0%)	0.00	0.00–>999.99	*p* = 0.989	0.00	0.00–>999.99	*p* = 0.989
	None	142	51 (35.9%)	Reference			Reference		
Status of constipation	Yes	111	40 (36.0%)	1.04	0.64–1.68	*p* = 0.878	1.12	0.68–1.83	*p* = 0.660
	No	83	29 (34.9%)	Reference			Reference		

*Adjusted for baseline PD medication use. HR, hazard ratio; CI, confidence interval; RBD, REM sleep behavior disorder; LED, L-dopa equivalent dose; UPDRS, Unified Parkinson’s Disease Rating Scale.

The results of the subsequent multivariate analyses of these factors are presented in Tables 4, 5. Among the factors tested, disease severity and time from the onset of motor symptoms to referral were significant in the multivariate analysis, suggesting a higher risk of worsening (defined as worsening of five or more points in the UPDRS III total score from baseline) in patients with milder severity and a longer time from the onset of motor symptoms to referral. Patients with a baseline UPDRS III score >20.0 had a lower risk of meeting the predefined worsening criterion than those with score ≤20.0 (HR, 0.39; 95% CI, 0.18–0.85). Additionally, patients who waited longer (2,359 days or more) from the onset of motor symptoms to their referral were 2.45 (95% CI: 1.23–4.89) times more likely to experience worsening than those who were referred sooner. Hyposmia was not a significant factor in the multivariate analysis, but the *p* value was close to 0.05 (*p* = 0.065), indicating a trend toward a higher risk of clinically relevant worsening in patients with hyposmia than for those without hyposmia (HR, 1.92; 95% CI, 0.96–3.83).

**Table 4:** Cox proportional hazards model (Multivariate analysis): Time to worsening of UPDRS III total score defined as a 5-point increase from baseline.

Variable	Category	*N*	With event (%)	Adjusted HR*	95% CI	Wald test
Total number of patients		194	69 (35.6%)			
Disease severity (Baseline UPDRS III Score)	≤Mean	112	49 (43.8%)	Reference		
	>Mean	82	20 (24.4%)	0.39	0.18–0.85	*p* = 0.017
Status of hyposmia	Yes	61	25 (41.0%)	1.92	0.96–3.83	*p* = 0.065
	No	68	14 (20.6%)	Reference		
Time from the onset of motor symptoms to referral to a specialized hospital (Juntendo University)	<Mean	119	36 (30.3%)	Reference		
	≥Mean	75	33 (44.0%)	2.45	1.23–4.89	*p* = 0.011
Status of orthostatic hypotension	Yes	44	22 (50.0%)	1.40	0.71–2.77	*p* = 0.331
	No	140	42 (30.0%)	Reference		

*Adjusted for baseline PD medication use. HR, hazard ratio; CI, confidence interval; UPDRS, Unified Parkinson’s Disease Rating Scale.

**Table 5:** Logistic regression model (Multivariate analysis): UPDRS III total score increased 5 points from baseline at 24 months.

Variable	Category	*N*	With event (%)	Adjusted OR*	95% CI	Wald test
Total number of patients		163	29 (17.8%)			
Disease severity (baseline UPDRS III score)	≤Mean	93	25 (26.9%)	Reference		
	>Mean	70	4 (5.7%)	0.15	0.05–0.48	*p* = 0.001
Status of orthostatic hypotension	Yes	33	10 (30.3%)	3.20	1.21–8.47	*p* = 0.019
	No	123	18(14.6%)	Reference		

*Adjusted for baseline PD medication use. OR, odds ratio; CI, confidence interval; UPDRS, Unified Parkinson’s Disease Rating Scale.

### 3.2 Logistic regression model analysis

The results of the univariate analysis for clinically relevant worsening, defined as a worsening of the UPDRS III score by five points from baseline, are presented in Table 6. Disease severity and OH status were significantly associated with this outcome after adjusting for the baseline PD medication use. The results of the subsequent multivariate analyses of these factors are presented in Table 5. Both disease severity and OH status were significant factors in the multivariate analysis. These results indicate a greater likelihood of meeting the predefined worsening criterion among patients with milder baseline severity and those with OH, independently of the other variables included in the model. Patients with a baseline UPDRS III score >20.0 had an adjusted OR of 0.15 (95% CI, 0.05–0.48), indicating a lower risk of worsening than those with a score at or below the mean. For patients with OH, the adjusted OR was 3.20 (95% CI, 1.21–8.47), indicating a higher likelihood of meeting the predefined worsening criterion compared to those without OH.

**Table 6:** Logistic regression model (univariate analysis): UPDRS III total score increased 5 points from baseline at 24 months.

Variable	Category	*N*	*N* (%)	With event (%)	95% CI	Wald test	Adjusted OR*	95% CI	Wald test
Total number of patients		163	29 (17.8%)						
Sex	Male	71	10 (14.1%)	Reference			Reference		
	Female	92	19 (20.7%)	1.59	0.69–3.67	*p* = 0.280	1.63	0.71–3.78	*p* = 0.252
Age	<Mean	70	9 (12.9%)	Reference			Reference		
	≥Mean	93	20 (21.5%)	1.86	0.79–4.38	*p* = 0.157	1.85	0.79–4.37	*p* = 0.159
Age at onset of PD	<50	26	3 (11.5%)	Reference			Reference		
	≥50	137	26 (19.0%)	1.80	0.50–6.44	*p* = 0.369	1.83	0.51–6.56	*p* = 0.354
Status of RBD	Yes	34	9 (26.5%)	1.96	0.80–4.82	*p* = 0.142	2.03	0.82–4.99	*p* = 0.125
	No	129	20 (15.5%)	Reference			Reference		
Daily L-dopa dose	≤Mean	83	16 (19.3%)	Reference			Reference		
	>Mean	80	13 (16.3%)	0.81	0.36–1.82	*p* = 0.614	0.79	0.35–1.77	*p* = 0.564
LED	≤Mean	93	17 (18.3%)	Reference			Reference		
	>Mean	67	11 (16.4%)	0.88	0.38–2.02	*p* = 0.760	—	—	—
Disease severity (baseline UPDRS III Score)	≤Mean	93	25 (26.9%)	Reference			Reference		
	>Mean	70	4 (5.7%)	0.16	0.05–0.50	*p* = 0.001	0.16	0.05–0.49	*p* = 0.001
Status of hyposmia	Yes	52	8 (15.4%)	1.48	0.48–4.62	*p* = 0.494	1.56	0.50–4.84	*p* = 0.446
	No	55	6 (10.9%)	Reference			Reference		
Time from the onset of motor symptoms to referral to a specialized hospital (Juntendo University)	<Mean	93	15 (16.1%)	Reference			Reference		
	≥Mean	70	14 (20.0%)	1.30	0.58–2.91	*p* = 0.523	1.27	0.57–2.84	*p* = 0.565
Status of orthostatic hypotension	Yes	33	10 (30.3%)	2.54	1.04–6.21	*p* = 0.042	2.63	1.07–6.46	*p* = 0.035
	No	123	18 (14.6%)	Reference			Reference		
Family history of Parkinson’s disease	First degree	7	2 (28.6%)	1.81	0.33–9.97	*p* = 0.956	1.79	0.32–9.87	*p* = 0.956
	Second degree	8	1 (12.5%)	0.65	0.08–5.54	*p* = 0.974	0.64	0.07–5.48	*p* = 0.974
	Third degree	8	1 (12.5%)	0.65	0.08–5.54	*p* = 0.974	0.64	0.07–5.48	*p* = 0.974
	Fourth degree	1	0 (0.0%)	<0.01	<0.01–>999.99	*p* = 0.967	<0.01	<0.01–>999.99	*p* = 0.967
	None	116	21 (18.1%)	Reference			Reference		
Status of constipation	Yes	92	16 (17.4%)	0.94	0.42–2.11	*p* = 0.879	0.91	0.40–2.04	*p* = 0.813
	No	71	13 (18.3%)	Reference			Reference		

*Adjusted for baseline PD medication use. OR, odds ratio; CI, confidence interval; RBD, REM sleep behavior disorder; LED, L-dopa equivalent dose; UPDRS, Unified Parkinson’s Disease Rating Scale.

**Figure 1:**
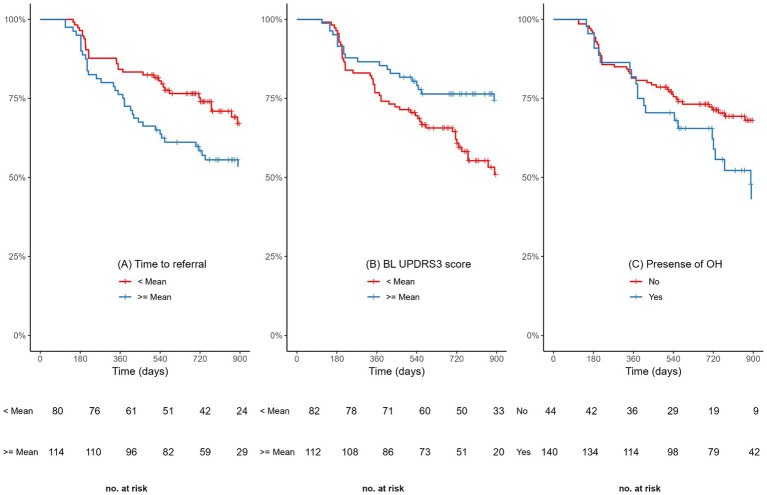
Kaplan–Meier curves of freedom from clinically relevant worsening, defined as worsening of UPDRS III score by 5 points from baseline, stratified by **(A)** time from motor symptom onset to referral to the site, **(B)** baseline (BL) UPDRS III score, and **(C)** presence of orthostatic hypotension (OH).

The Kaplan–Meier curves stratified by time to referral, baseline UPDRS III score, and OH status are shown in Figure 1. The outcomes of the univariate and subsequent multivariate analyses using alternative definitions of worsening outcomes are presented in Supplementary Tables S2–S10 (Cox proportional hazards model) and Supplementary Tables S11–S15 (logistic regression model) in Supplementary material.

## 4 Discussion

This was a longitudinal observational study of patients with PD receiving standard therapy from neurology specialists in routine clinical settings without any specific restrictions. This study aimed to evaluate the longitudinal clinical course and factors associated with clinically relevant worsening. Taken together, our findings suggest that (i) motor symptoms may transiently improve following referral to specialized care, (ii) baseline motor severity and time to referral are associated with short-term clinically relevant worsening, and (iii) symptomatic orthostatic hypotension may serve as a practical marker of vulnerability even in patients with predominantly mild to moderate PD.

The majority of the study population had mild to moderate disease severity, with HY stage 1 or 2 in 55.7% and HY stage 3 in 28.4%, whereas only 6.2% had advanced disease corresponding to HY stage 4 or 5 (15). Only a few patients in our study exhibited motor complications at entry (two patients with a total UPDRS IV score of 2 and one patient with a score of 4).

Notably, the UPDRS III score improved during the initial 6-month period, with a mean (SD) change of 3.3 (8.2) points. This finding suggests that further symptomatic improvement may be achievable after referral to specialized care, even among patients who had already received treatment for a median of more than 4 years from the onset of motor symptoms. Reinoso et al. also reported an initial improvement in motor scores during the first 2 years, followed by a return to baseline after 2–2.5 years, relative stability for up to 7 years, and subsequent progression (8). In our cohort, the initial improvement in mean UPDRS III score was maintained throughout the observation period of up to 2.5 years and did not return to baseline. This difference may partly reflect the relatively short observation period in our study, which may have been insufficient to capture the later stability and progression phases described by Reinoso et al. In addition, whereas Reinoso et al. included patients assessed within 2 years of diagnosis, the mean disease duration at baseline in our study was 6.22 years. These differences in disease duration and study design limit direct comparability between the two studies, and longer follow-up will be required to characterize the long-term clinical course of PD in Japanese patients.

We explored potential factors associated with clinically relevant worsening. For the representative outcome, defined as a worsening of the UPDRS III score by five points or more from the baseline, baseline disease severity was consistently identified as a significant factor in both multivariate models. Time from PD onset to referral to Juntendo University Hospital was significant only in the Cox proportional hazards model, whereas symptomatic OH was significant only in the logistic regression model.

This study indicated a higher risk of clinically relevant worsening in patients with milder baseline motor symptoms, as reflected by a lower baseline UPDRS III score. This finding suggests that patients with less severe baseline motor impairment may be more likely to meet the predefined worsening criterion, which is consistent with previous reports by Post (16), Reinoso (8), and findings from the Parkinson’s Progression Marker Initiative (PPMI) study cohort reported by Chahine (10).

A longer time from PD onset to referral to Juntendo University Hospital was also associated with a higher risk of clinically relevant worsening. This may appear inconsistent with the finding that patients with milder baseline motor symptoms had a higher risk of subsequent worsening. Notably, however, the mean baseline UPDRS III score was higher in patients with a longer time to referral than in those with a shorter time to referral (26.1 vs. 16.2, respectively). These results suggest that the association between baseline motor severity, referral timing, and subsequent worsening is complex and may be influenced by differences in baseline disease status, referral patterns, and prior treatment before specialist evaluation. The association between longer time to referral and higher baseline motor impairment is consistent with the potential clinical importance of timely access to specialized care in PD. However, caution is warranted in interpreting this measure, as the period prior to specialist treatment does not necessarily reflect the duration of treatment-naïve status. Additionally, our data do not clarify how patients were managed by their previous physicians or whether their treatment was modified upon referral to Juntendo University Hospital.

Orthostatic hypotension is a well-known autonomic symptom affecting approximately 30%–65% of patients with PD (17, 18), with its prevalence increasing over time (19). OH has been recognized as a part of the diffuse/malignant PD phenotype (9, 20), and is associated with reduced daily functioning and an increased risk of injurious falls (21, 22). OH in PD has been associated with poorer long-term outcomes, including cognitive decline and mortality (23–25). Consistent with these reports, our study showed a higher risk of clinically relevant worsening in patients with symptomatic OH. The association between OH and worsening should not be considered confirmatory because of the discrepancy between the two analytical models. However, the clear separation observed in the Kaplan–Meier curve between patients with and without OH suggests a possible trend toward a higher risk of this outcome among patients with symptomatic OH. Thus, symptomatic OH may represent a potential prognostic marker in patients with predominantly mild to moderate PD, despite the relatively short follow-up period of 18–30 months. Importantly, OH was assessed based on medical interviews, meaning that only symptomatic patients were classified as having OH. Although this approach may have underestimated the true prevalence of OH, particularly asymptomatic hemodynamic OH, symptomatic OH can be readily identified in routine clinical practice. Therefore, patient-reported symptoms suggestive of OH may still provide practical information for risk stratification. In this study, the mean baseline UPDRS III score was comparable between patients with and without symptomatic OH (19.0 vs. 19.7, respectively), suggesting that symptomatic OH may help identify vulnerable patients beyond baseline motor severity alone.

Other autonomic symptoms such as RBD, hyposmia, and constipation were not significantly associated with any event across the statistical models. This may partly reflect the limited sample size and use of routine medical interviews rather than objective testing (e.g., Sniffin’ Sticks test for hyposmia). Therefore, these symptoms, as well as asymptomatic OH, may have been underdiagnosed, and the lack of significant associations should be interpreted cautiously. Among the autonomic symptoms assessed in routine interviews, symptomatic OH may have been the most clinically detectable marker associated with short-term worsening in this cohort.

We intentionally used two complementary models to explore factors associated with clinically relevant worsening, reflecting the exploratory nature of this study. The Cox proportional hazards model incorporated time-to-event information, whereas the 24-month landmark logistic regression model evaluated associations at a fixed time point. We interpreted factors identified consistently across both models as more robust signals, while factors identified in only one model were regarded as hypothesis-generating findings requiring further validation.

For the representative event of UPDRS III worsening of five points or more, only baseline disease severity was significant in both models. In contrast, time from PD onset to referral to the Juntendo University Hospital was significant only in the Cox model, whereas symptomatic OH was significant only in the logistic regression model. These discrepancies likely reflect differences in model structure and event inclusion. Specifically, the Cox model included time-to-event information and 69 events, whereas the 24-month landmark logistic regression model included 29 events at the fixed landmark time point.

An increase of five points or more in UPDRS III has been reported to represent a clinically important worsening, consistent with previous studies on minimal clinically important difference (26). Our results suggest that this criterion is sensitive enough to explore patient characteristics associated with short-term worsening within a relatively short observation period. Similar analyses were performed using other exploratory worsening outcomes (worsening of UPDRS III score with other cut-off values, worsening of HY stage, increases in the daily dose of PD medications, and worsening of UPDRS IV score). The detailed outcomes are shown in Supplementary material. Across these analyses, several motor-related definitions, including worsening of UPDRS III score and worsening of HY stage, supported the observation that baseline disease severity and time from PD onset to referral were associated with clinically relevant worsening.

When treatment escalation was analyzed using doubling of the LED as the outcome in the Cox proportional hazards model, only baseline L-dopa dose was significantly associated with this event. A lower baseline dose was associated with a higher likelihood of LED doubling. Given that the baseline L-dopa dose was relatively low in some patients, doubling of LED may reflect dose adjustment after referral rather than clinical worsening itself.

Because motor complications require careful monitoring in patients receiving L-dopa, particularly in younger patients who are at higher risk, an increase of two or more points in the UPDRS IV was evaluated as an indicator of clinically relevant worsening related to motor complications, such as wearing-off or dyskinesia. In the multivariate Cox proportional hazards model, only time to referral to Juntendo University Hospital remained significant. Other baseline characteristics, including age, baseline motor severity, and L-dopa dose, were significant only in univariate analyses. The survival curve for the entire population (Supplementary Figure S2) showed a steep increase in this event around 1 year, whereas approximately 90% of patients remained event-free before that time point.

Our study has several limitations. First, because this was an observational cohort study, selection bias cannot be excluded. Of the 348 patients in the database, 194 were included in the analysis. Baseline demographics and disease characteristics were compared between the analyzed population and the overall database population, and no clinically meaningful differences were identified (Supplementary Table S16). Therefore, although residual selection bias cannot be excluded, its impact on the main findings is likely limited. Second, all data were obtained from a single site in Japan, which may limit the generalizability of the results. Third, autonomic symptoms were assessed through medical interviews rather than objective testing and may therefore have been underdiagnosed. In particular, asymptomatic hemodynamic OH, RBD, and hyposmia may not have been fully captured, potentially leading to misclassification and underestimation of their associations with clinically relevant worsening. Fourth, because no universally accepted short-term definition of PD progression exists in this context, the thresholds used in this study should be interpreted as exploratory indicators of clinically relevant worsening rather than definitive progression criteria. In addition, outcomes related to motor fluctuations, dyskinesias, levodopa dosing frequency, and initiation of advanced device-aided therapies were not comprehensively assessed, limiting the interpretation of treatment escalation and symptomatic worsening. Finally, item-level UPDRS data were available only for a limited number of patients, restricting our ability to analyze motor subtypes such as tremor-dominant or postural instability and gait difficulty phenotypes.

Motor symptoms were evaluated using the original UPDRS rather than the MDS-UPDRS because the original UPDRS was routinely used at the study site during the study period. Because the two instruments differ in item composition, scoring structure, and clinimetric properties, direct numerical comparisons with studies using the MDS-UPDRS should be interpreted with caution. However, our analyses focused on within-cohort longitudinal changes and worsening outcomes based on the same instrument applied consistently across visits. Therefore, the impact of this discrepancy on our main conclusions is likely limited. Accordingly, we avoided direct numerical comparisons between UPDRS Part III and MDS-UPDRS Part III scores across studies.

## 5 Conclusion

Our study identified baseline disease severity, time to referral to specialized care, and symptomatic OH as potential factors associated with a higher risk of clinically relevant worsening. Notably, these associations were observed within a relatively short observation period of 18–30 months. Our findings suggest that a five-point worsening of the UPDRS III total score may serve as a clinically meaningful and practical measure for the exploratory assessment of short-term worsening. The association between longer time from PD onset to referral and clinically relevant worsening supports the potential importance of early diagnosis and timely referral to specialized care. Strategies that facilitate earlier access to specialized care may help optimize symptom management and identify patients at higher risk of clinical worsening.

In addition, the clinical course and potential factors associated with clinically relevant worsening observed in this Japanese cohort were broadly consistent with findings from studies conducted outside Japan, suggesting comparability in disease characteristics between Japanese and non-Japanese patients with PD. Larger prospective studies with longer follow-up are needed to characterize the clinical course of PD in Japan and to validate the potential factors associated with clinically relevant worsening identified in this study.

## Data Availability

The raw data supporting the conclusions of this article will be made available by the authors, without undue reservation.

## References

[1] Kalia LV Lang AE. Parkinson disease in 2015: evolving basic, pathological and clinical concepts in PD. Nat Rev Neurol. (2016) 12:65–6. doi: 10.1038/nrneurol.2015.249, 26782330

[2] Berardelli A Wenning GK Antonini A Berg D Bloem BR Bonifati V . EFNS/MDS-ES/ENS [corrected] recommendations for the diagnosis of Parkinson’s disease. Eur J Neurol. (2013) 20:16–34. doi: 10.1111/ene.12022, [published correction appears in Eur J Neurol 2013 20(2):406. doi:10.1111/ene.12111] 23279440

[3] Postuma RB Berg D Stern M Poewe W Olanow CW Oertel W . MDS clinical diagnostic criteria for Parkinson’s disease. Mov Disord. (2015) 30:1591–601. doi: 10.1002/mds.26424, 26474316

[4] Goetz CG. The history of Parkinson’s disease: early clinical descriptions and neurological therapies. Cold Spring Harb Perspect Med. (2011) 1:a008862. doi: 10.1101/cshperspect.a008862, 22229124 PMC3234454

[5] Poewe W Seppi K Tanner CM Halliday GM Brundin P . Parkinson disease. Nat Rev Dis Primers. (2017) 3:17013. doi: 10.1038/nrdp.2017.13, 28332488

[6] Parkinson’s Foundation. Understanding Parkinson’s Statistics. Miami, FL: Parkinson’s Foundation (2023).

[7] Yamawaki M Kusumi M Kowa H Nakashima K. Changes in prevalence and incidence of Parkinson’s disease in Japan during a quarter of a century. Neuroepidemiology. (2009) 32:263–9. doi: 10.1159/000201565, 19209006

[8] Reinoso G Allen JC Jr Au WL Seah SH Tay KY Tan LC. Clinical evolution of Parkinson’s disease and prognostic factors affecting motor progression: 9-year follow-up study. Eur J Neurol. (2015) 22:457–63. doi: 10.1111/ene.12476, 24888502

[9] Fereshtehnejad SM Romenets SR Anang JB Latreille V Gagnon JF Postuma RB. New clinical subtypes of Parkinson disease and their longitudinal progression: a prospective cohort comparison with other phenotypes. JAMA Neurol. (2015) 72:863–73. doi: 10.1001/jamaneurol.2015.0703 26076039

[10] Chahine LM Siderowf A Barnes J Seedorff N Caspell-Garcia C Simuni T . Predicting progression in Parkinson’s disease using baseline and 1-year change measures. J Parkinsons Dis. (2019) 9:665–79. doi: 10.3233/JPD-181518, 31450510 PMC6839498

[11] Sato K Hatano T Yamashiro K Kagohashi M Nishioka K Izawa N . Prognosis of Parkinson’s disease: time to stage III, IV, V, and to motor fluctuations. Mov Disord. (2006) 21:1384–95. doi: 10.1002/mds.20993, 16763980

[12] Yoritaka A Shimo Y Hatano T Hattori N. Motor/nonmotor symptoms and progression in patients with Parkinson’s disease: prevalence and risks in a longitudinal study. Parkinsons Dis. (2020) 2020:2735361. doi: 10.1155/2020/2735361, 32655850 PMC7322581

[13] Breslow NE. Discussion of professor cox’s paper. JR Stat Soc B. (1972) 34:216–7.

[14] Mehanna R Smilowska K Fleisher J Post B Hatano T Pimentel Piemonte ME . Age cutoff for early-onset Parkinson’s disease: recommendations from the international Parkinson and movement disorder society task force on early onset Parkinson’s disease. Mov Disord Clin Pract. (2022) 9:869–78. doi: 10.1002/mdc3.13523, 36247919 PMC9547138

[15] Hoehn MM Yahr MD. Parkinsonism: onset, progression and mortality. Neurology. (1967) 17:427–42. doi: 10.1212/wnl.17.5.427, 6067254

[16] Post B Merkus MP de Haan RJ CARPA Study Group. Prognostic factors for the progression of Parkinson’s disease: a systematic review. Mov Disord. (2007) 22:quiz1988:1839–51. doi: 10.1002/mds.21537 17595026

[17] Velseboer DC de Haan RJ Wieling W Goldstein DS de Bie RM. Prevalence of orthostatic hypotension in Parkinson’s disease: a systematic review and meta-analysis. Parkinsonism Relat Disord. (2011) 17:724–9. doi: 10.1016/j.parkreldis.2011.04.016, 21571570 PMC5199613

[18] Hiorth YH Pedersen KF Dalen I Tysnes OB Alves G. Orthostatic hypotension in Parkinson disease: a 7-year prospective population-based study. Neurology. (2019) 93:e1526–34. doi: 10.1212/WNL.0000000000008314, 31527282

[19] Beach P McKay JL. Longitudinal prevalence of neurogenic orthostatic hypotension in the idiopathic Parkinson progression marker initiative (PPMI) cohort. Auton Neurosci. (2024) 253:103173. doi: 10.1016/j.autneu.2024.103173, 38692034 PMC11128342

[20] Merola A Romagnolo A Dwivedi AK Padovani A Berg D Garcia-Ruiz PJ . Benign versus malignant Parkinson disease: the unexpected silver lining of motor complications. J Neurol. (2020) 267:2949–60. doi: 10.1007/s00415-020-09954-6, 32488298

[21] Romagnolo A Zibetti M Merola A Canova D Sarchioto M Montanaro E . Cardiovascular autonomic neuropathy and falls in Parkinson disease: a prospective cohort study. J Neurol. (2019) 266:85–91. doi: 10.1007/s00415-018-9104-4, 30382389

[22] Merola A Romagnolo A Rosso M Suri R Berndt Z Maule S . Autonomic dysfunction in Parkinson’s disease: a prospective cohort study. Mov Disord. (2018) 33:391–7. doi: 10.1002/mds.27268, 29278286

[23] Kotagal V Lineback C Bohnen NI Albin RL Calm PD Parkinson Study Group. Orthostatic hypotension predicts motor decline in early Parkinson disease. Parkinsonism Relat Disord. (2016) 32:127–9. doi: 10.1016/j.parkreldis.2016.09.011, 27639815 PMC5114666

[24] Ruiz Barrio I Miki Y Jaunmuktane ZT Warner T De Pablo-Fernandez E. Association between orthostatic hypotension and dementia in patients with Parkinson disease and multiple system atrophy. Neurology. (2023) 100:e998–e1008. doi: 10.1212/WNL.0000000000201659, 36526431 PMC9990860

[25] Goldstein DS Holmes C Sharabi Y Wu T. Survival in synucleinopathies: a prospective cohort study. Neurology. (2015) 85:1554–61. doi: 10.1212/WNL.0000000000002086, 26432848 PMC4642141

[26] Hauser RA Gordon MF Mizuno Y Poewe W Barone P Schapira AH . Minimal clinically important difference in Parkinson’s disease as assessed in pivotal trials of pramipexole extended release. Parkinsons Dis. (2014) 2014:467131. doi: 10.1155/2014/467131, 24800101 PMC3995302

[27] De Pablo-Fernandez E Tur C Revesz T Lees AJ Holton JL Warner TT. Association of autonomic dysfunction with disease progression and survival in Parkinson disease. JAMA Neurol. (2017) 74:970–6. doi: 10.1001/jamaneurol.2017.1125, 28655059 PMC5710320

