# Supplementary material for: Exploration of the longitudinal clinical course and potential prognostic factors in Parkinson’s disease: 2.5-year observational study in Japan
Source: Front Neurol. 2026 May 26;17:1845594. doi: 10.3389/fneur.2026.1845594 (PMC13246703; doi:10.3389/fneur.2026.1845594)
Supplement: Supplementary file 1 [file Table_1.DOCX]

**Supplement 1：Supplementary Tables and Figures**

Table S1. Definitions of clinically relevant worsening outcome and number of patients who met each criterion.

| Endpoint | N (%) |
| --- | --- |
| ≥ 25% worsening in UPDRS score | 77 (39.7) |
| ≥ 5-point worsening in UPDRS score | 69 (35.6) |
| ≥ 1-point worsening in Hoehn-Yahr score | 70 (36.1) |
| ≥ 1.5-fold increment in L-dopa dose | 34 (17.5) |
| ≥ 2-fold increment in LED | 15 (7.7) |
| ≥ 3-fold increment in LED | 8 (4.1) |
| UPDRS IV score reaching ≥ 2-point | 136 (70.1) |

Table S2. Cox proportional hazard model (Univariate analysis): Time to worsening of UPDRS III total score defined as 25% increase from baseline

|  |  |  | N (%) | | HR | HR (95%CI) | Wald test | HR adjusted* | HR adjusted (95%CI) | Wald test |
| --- | --- | --- | --- | --- | --- | --- | --- | --- | --- | --- |
| Total number of patients |  | 194 | 77 | (39.7%) |  |  |  |  |  |  |
| Gender | Male | 87 | 34 | (39.1%) | Reference |  |  | Reference |  |  |
|  | Female | 107 | 43 | (40.2%) | 1.01 | 0.64-1.59 | *p=*0.955 | 0.98 | 0.62-1.54 | *p=*0.923 |
| Age | <Mean | 87 | 33 | (37.9%) | Reference |  |  | Reference |  |  |
|  | ≥Mean | 107 | 44 | (41.1%) | 0.97 | 0.62-1.53 | *p=*0.907 | 0.99 | 0.63-1.55 | *p=*0.952 |
|  | Unknown | 0 | 0 | - |  |  |  |  |  |  |
| Age at onset of PD | <50 | 29 | 11 | (37.9%) | Reference |  |  | Reference |  |  |
|  | ≥50 | 165 | 66 | (40.0%) | 0.96 | 0.51-1.83 | *p=*0.912 | 0.94 | 0.50-1.79 | *p=*0.859 |
|  | Unknown | 0 | 0 | - |  |  |  |  |  |  |
| RBD | Yes | 41 | 17 | (41.5%) | 1.09 | 0.64-1.88 | *p=*0.744 | 1.07 | 0.62-1.84 | *p=*0.807 |
|  | No | 153 | 60 | (39.2%) | Reference |  |  | Reference |  |  |
|  | Not Evaluable | 0 | 0 | - |  |  |  |  |  |  |
|  | Unknown | 0 | 0 | - |  |  |  |  |  |  |
| Daily L-dopa dose | ≤Mean | 105 | 41 | (39.0%) | Reference |  |  | Reference |  |  |
|  | >Mean | 89 | 36 | (40.4%) | 0.97 | 0.62-1.53 | *p=*0.905 | 1.01 | 0.64-1.59 | *p=*0.973 |
|  | Unknown | 0 | 0 | - |  |  |  |  |  |  |
| LED | <=Mean | 112 | 44 | (39.3%) | Reference |  |  | Reference |  |  |
|  | >Mean | 77 | 29 | (37.7%) | 0.94 | 0.59-1.51 | *p=*0.799 | - | - | - |
|  | Unknown | 5 | 4 | (80.0%) |  |  |  |  |  |  |
| Disease severity （Baseline UPDRS III Score） | ≤Mean | 112 | 65 | (58.0%) | Reference |  |  | Reference |  |  |
|  | >Mean | 82 | 12 | (14.6%) | 0.18 | 0.09-0.33 | *p*<0.001 | 0.17 | 0.09-0.32 | *p*<0.001 |
|  | Unknown | 0 | 0 | - |  |  |  |  |  |  |
| Hyposmia | Yes | 61 | 25 | (41.0%) | 1.36 | 0.76-2.42 | *p=*0.305 | 1.26 | 0.70-2.28 | *p=*0.444 |
|  | No | 68 | 21 | (30.9%) | Reference |  |  | Reference |  |  |
|  | Not Evaluable | 0 | 0 | - |  |  |  |  |  |  |
|  | Unknown | 65 | 31 | (47.7%) |  |  |  |  |  |  |
| Time from the onset of motor symptoms to referral to specialized care (Juntendo University Hospital) | <Mean | 119 | 47 | (39.5%) | Reference |  |  | Reference |  |  |
|  | ≥Mean | 75 | 30 | (40.0%) | 0.97 | 0.61-1.54 | *p=*0.897 | 1.00 | 0.63-1.59 | *p=*0.998 |
|  | Unknown | 0 | 0 | - |  |  |  |  |  |  |
| Orthostatic hypotension | Yes | 44 | 22 | (50.0%) | 1.49 | 0.90-2.45 | *p=*0.121 | 1.52 | 0.92-2.51 | *p=*0.102 |
|  | No | 140 | 51 | (36.4%) | Reference |  |  | Reference |  |  |
|  | Not Evaluable | 1 | 0 | (0.0%) |  |  |  |  |  |  |
|  | Unknown | 9 | 4 | (44.4%) |  |  |  |  |  |  |
| Family history of Parkinson’s disease | First degree | 7 | 4 | (57.1%) | 1.15 | 0.42-3.18 | *p=*0.785 | 1.16 | 0.42-3.21 | *p=*0.769 |
|  | Second degree | 9 | 5 | (55.6%) | 1.23 | 0.49-3.09 | *p=*0.662 | 1.24 | 0.49-3.12 | *p=*0.645 |
|  | Third degree | 9 | 1 | (11.1%) | 0.19 | 0.03-1.41 | *p=*0.105 | 0.20 | 0.03-1.43 | *p=*0.107 |
|  | Fourth degree | 1 | 0 | (0.0%) | 0.00 | 0.00->999.99 | *p=*0.987 | 0.00 | 0.00->999.99 | *p=*0.987 |
|  | None | 142 | 58 | (40.8%) | Reference |  |  | Reference |  |  |
|  | Unknown | 26 | 9 | (34.6%) |  |  |  |  |  |  |
| Constipation | Yes | 111 | 39 | (35.1%) | 0.67 | 0.43-1.05 | *p=*0.082 | 0.70 | 0.44-1.10 | *p=*0.118 |
|  | No | 83 | 38 | (45.8%) | Reference |  |  | Reference |  |  |
|  | Not Evaluable | 0 | 0 | - |  |  |  |  |  |  |
|  | Unknown | 0 | 0 | - |  |  |  |  |  |  |

* adjusted for baseline PD medication use. HR, hazard ratio; CI, confidence interval; RBD, REM sleep behavior disorder; LED, L-dopa equivalent dose; UPDRS, Unified Parkinson’s Disease Rating Scale

Table S3. Cox proportional hazard model (Univariate analysis): Time to worsening of PD symptom defined by 1.5 holds L-dopa dose from baseline

|  |  |  | N (%) | | HR | HR (95%CI) | Wald test | HR adjusted* | HR adjusted (95%CI) | Wald test |
| --- | --- | --- | --- | --- | --- | --- | --- | --- | --- | --- |
| Total number of patients |  | 194 | 34 | (17.5%) |  |  |  |  |  |  |
| Gender | Male | 87 | 16 | (18.4%) | Reference |  |  | Reference |  |  |
|  | Female | 107 | 18 | (16.8%) | 0.88 | 0.45-1.72 | *p=*0.706 | 0.90 | 0.46-1.76 | *p=*0.758 |
| Age | <Mean | 87 | 13 | (14.9%) | Reference |  |  | Reference |  |  |
|  | ≥Mean | 107 | 21 | (19.6%) | 1.25 | 0.63-2.51 | *p=*0.520 | 1.24 | 0.62-2.47 | *p=*0.545 |
|  | Unknown | 0 | 0 | - |  |  |  |  |  |  |
| Age at onset of PD | <50 | 29 | 3 | (10.3%) | Reference |  |  | Reference |  |  |
|  | ≥50 | 165 | 31 | (18.8%) | 1.90 | 0.58-6.20 | *p=*0.290 | 1.93 | 0.59-6.30 | *p=*0.279 |
|  | Unknown | 0 | 0 | - |  |  |  |  |  |  |
| RBD | Yes | 41 | 11 | (26.8%) | 1.90 | 0.93-3.91 | *p=*0.079 | 1.92 | 0.94-3.94 | *p=*0.075 |
|  | No | 153 | 23 | (15.0%) | Reference |  |  | Reference |  |  |
|  | Not Evaluable | 0 | 0 | - |  |  |  |  |  |  |
|  | Unknown | 0 | 0 | - |  |  |  |  |  |  |
| Daily L-dopa dose | ≤Mean | 105 | 27 | (25.7%) | Reference |  |  | Reference |  |  |
|  | >Mean | 89 | 7 | (7.9%) | 0.24 | 0.10-0.55 | *p*<0.001 | 0.23 | 0.10-0.54 | *p*<0.001 |
|  | Unknown | 0 | 0 | - |  |  |  |  |  |  |
| LED | ≤Mean | 112 | 25 | (22.3%) | Reference |  |  | Reference |  |  |
|  | >Mean | 77 | 9 | (11.7%) | 0.45 | 0.21-0.96 | *p=*0.039 | - | - | - |
|  | Unknown | 5 | 0 | (0.0%) |  |  |  |  |  |  |
| Disease severity （Baseline UPDRS III Score） | ≤Mean | 112 | 22 | (19.6%) | Reference |  |  | Reference |  |  |
|  | >Mean | 82 | 12 | (14.6%) | 0.70 | 0.35-1.41 | *p=*0.317 | 0.69 | 0.34-1.39 | *p=*0.301 |
|  | Unknown | 0 | 0 | - |  |  |  |  |  |  |
| Hyposmia | Yes | 61 | 12 | (19.7%) | 1.37 | 0.59-3.16 | *p=*0.466 | 1.41 | 0.61-3.27 | *p=*0.419 |
|  | No | 68 | 10 | (14.7%) | Reference |  |  | Reference |  |  |
|  | Not Evaluable | 0 | 0 | - |  |  |  |  |  |  |
|  | Unknown | 65 | 12 | (18.5%) |  |  |  |  |  |  |
| Time from the onset of motor symptoms to referral to specialized care (Juntendo University Hospital) | <Mean | 119 | 18 | (15.1%) | Reference |  |  | Reference |  |  |
|  | ≥Mean | 75 | 16 | (21.3%) | 1.32 | 0.67-2.59 | *p=*0.423 | 1.29 | 0.66-2.53 | *p=*0.461 |
|  | Unknown | 0 | 0 | - |  |  |  |  |  |  |
| Orthostatic hypotension | Yes | 44 | 10 | (22.7%) | 1.53 | 0.72-3.23 | *p=*0.266 | 1.57 | 0.74-3.31 | *p=*0.241 |
|  | No | 140 | 22 | (15.7%) | Reference |  |  | Reference |  |  |
|  | Not Evaluable | 1 | 0 | (0.0%) |  |  |  |  |  |  |
|  | Unknown | 9 | 2 | (22.2%) |  |  |  |  |  |  |
| Family history of Parkinson’s disease | First degree | 7 | 1 | (14.3%) | 0.81 | 0.11-6.01 | *p=*0.839 | 0.80 | 0.11-5.94 | *p=*0.830 |
|  | Second degree | 9 | 2 | (22.2%) | 1.49 | 0.35-6.30 | *p=*0.589 | 1.47 | 0.35-6.22 | *p=*0.601 |
|  | Third degree | 9 | 1 | (11.1%) | 0.61 | 0.08-4.54 | *p=*0.633 | 0.61 | 0.08-4.48 | *p=*0.624 |
|  | Fourth degree | 1 | 0 | (0.0%) | 0.00 | 0.00->999.99 | *p=*0.989 | 0.00 | 0.00->999.99 | *p=*0.993 |
|  | None | 142 | 24 | (16.9%) | Reference |  |  | Reference |  |  |
|  | Unknown | 26 | 6 | (23.1%) |  |  |  |  |  |  |
| Constipation | Yes | 111 | 19 | (17.1%) | 0.92 | 0.47-1.81 | *p=*0.803 | 0.89 | 0.45-1.75 | *p=*0.732 |
|  | No | 83 | 15 | (18.1%) | Reference |  |  | Reference |  |  |
|  | Not Evaluable | 0 | 0 | - |  |  |  |  |  |  |
|  | Unknown | 0 | 0 | - |  |  |  |  |  |  |

* adjusted for baseline PD medication use. HR, hazard ratio; CI, confidence interval; RBD, REM sleep behavior disorder; LED, L-dopa equivalent dose; UPDRS, Unified Parkinson’s Disease Rating Scale

Table S4. Cox proportional hazard model (Univariate analysis): Time to worsening of PD symptom defined as doubling of LED from baseline

|  |  |  | N (%) | | HR | HR (95%CI) | Wald test | HR adjusted* | HR adjusted (95%CI) | Wald test |
| --- | --- | --- | --- | --- | --- | --- | --- | --- | --- | --- |
| Total number of patients |  | 194 | 15 | (7.7%) |  |  |  |  |  |  |
| Gender | Male | 87 | 11 | (12.6%) | Reference |  |  | Reference |  |  |
|  | Female | 107 | 4 | (3.7%) | 0.28 | 0.09-0.88 | *p=*0.030 | 0.29 | 0.09-0.91 | *p=*0.034 |
| Age | <Mean | 87 | 9 | (10.3%) | Reference |  |  | Reference |  |  |
|  | ≥Mean | 107 | 6 | (5.6%) | 0.51 | 0.18-1.43 | *p=*0.199 | 0.50 | 0.18-1.41 | *p=*0.190 |
|  | Unknown | 0 | 0 | - |  |  |  |  |  |  |
| Age at onset of PD | <50 | 29 | 4 | (13.8%) | Reference |  |  | Reference |  |  |
|  | ≥50 | 165 | 11 | (6.7%) | 0.45 | 0.14-1.43 | *p=*0.177 | 0.46 | 0.15-1.45 | *p=*0.187 |
|  | Unknown | 0 | 0 | - |  |  |  |  |  |  |
| RBD | Yes | 41 | 3 | (7.3%) | 0.92 | 0.26-3.25 | *p=*0.895 | 0.93 | 0.26-3.29 | *p=*0.908 |
|  | No | 153 | 12 | (7.8%) | Reference |  |  | Reference |  |  |
|  | Not Evaluable | 0 | 0 | - |  |  |  |  |  |  |
|  | Unknown | 0 | 0 | - |  |  |  |  |  |  |
| Daily L-dopa dose | ≤Mean | 105 | 13 | (12.4%) | Reference |  |  | Reference |  |  |
|  | >Mean | 89 | 2 | (2.2%) | 0.17 | 0.04-0.74 | *p=*0.019 | 0.16 | 0.04-0.72 | *p=*0.017 |
|  | Unknown | 0 | 0 | - |  |  |  |  |  |  |
| LED | ≤Mean | 112 | 14 | (12.5%) | Reference |  |  | Reference |  |  |
|  | >Mean | 77 | 1 | (1.3%) | 0.10 | 0.01-0.74 | *p=*0.024 | - | - | - |
|  | Unknown | 5 | 0 | (0.0%) |  |  |  |  |  |  |
| Disease severity （Baseline UPDRS III Score） | ≤Mean | 112 | 5 | (4.5%) | Reference |  |  | Reference |  |  |
|  | >Mean | 82 | 10 | (12.2%) | 2.83 | 0.97-8.29 | *p=*0.057 | 2.81 | 0.96-8.22 | *p=*0.059 |
|  | Unknown | 0 | 0 | - |  |  |  |  |  |  |
| Hyposmia | Yes | 61 | 7 | (11.5%) | 4.11 | 0.85-19.81 | *p=*0.078 | 4.26 | 0.88-20.50 | *p=*0.071 |
|  | No | 68 | 2 | (2.9%) | Reference |  |  | Reference |  |  |
|  | Not Evaluable | 0 | 0 | - |  |  |  |  |  |  |
|  | Unknown | 65 | 6 | (9.2%) |  |  |  |  |  |  |
| Time from the onset of motor symptoms to referral to specialized care (Juntendo University Hospital) | <Mean | 119 | 9 | (7.6%) | Reference |  |  | Reference |  |  |
|  | ≥Mean | 75 | 6 | (8.0%) | 1.04 | 0.37-2.93 | *p=*0.936 | 1.02 | 0.36-2.86 | *p=*0.973 |
|  | Unknown | 0 | 0 | - |  |  |  |  |  |  |
| Orthostatic hypotension | Yes | 44 | 4 | (9.1%) | 1.30 | 0.41-4.16 | *p=*0.654 | 1.33 | 0.42-4.23 | *p=*0.632 |
|  | No | 140 | 10 | (7.1%) | Reference |  |  | Reference |  |  |
|  | Not Evaluable | 1 | 0 | (0.0%) |  |  |  |  |  |  |
|  | Unknown | 9 | 1 | (11.1%) |  |  |  |  |  |  |
| Family history of Parkinson’s disease | First degree | 7 | 2 | (28.6%) | 6.11 | 1.30-28.84 | *p=*0.022 | 6.04 | 1.28-28.47 | *p=*0.023 |
|  | Second degree | 9 | 0 | (0.0%) | 0.00 | 0.00->999.99 | *p=*0.993 | 0.00 | 0.00->999.99 | *p=*0.993 |
|  | Third degree | 9 | 1 | (11.1%) | 2.12 | 0.26-16.91 | *p=*0.480 | 2.09 | 0.26-16.70 | *p=*0.488 |
|  | Fourth degree | 1 | 0 | (0.0%) | 0.00 | 0.00->999.99 | *p=*0.998 | 0.00 | 0.00->999.99 | *p=*0.998 |
|  | None | 142 | 8 | (5.6%) | Reference |  |  | Reference |  |  |
|  | Unknown | 26 | 4 | (15.4%) |  |  |  |  |  |  |
| Constipation | Yes | 111 | 8 | (7.2%) | 0.84 | 0.30-2.31 | *p=*0.730 | 0.81 | 0.29-2.23 | *p=*0.679 |
|  | No | 83 | 7 | (8.4%) | Reference |  |  | Reference |  |  |
|  | Not Evaluable | 0 | 0 | - |  |  |  |  |  |  |
|  | Unknown | 0 | 0 | - |  |  |  |  |  |  |

* adjusted for baseline PD medication use. HR, hazard ratio; CI, confidence interval; RBD, REM sleep behavior disorder; LED, L-dopa equivalent dose; UPDRS, Unified Parkinson’s Disease Rating Scale

Table S5. Cox proportional hazard model (Univariate analysis): Time to worsening of PD symptom defined as tripling of LED from baseline

|  |  |  | N (%) | | HR | HR (95%CI) | Wald test | HR adjusted* | HR adjusted (95%CI) | Wald test |
| --- | --- | --- | --- | --- | --- | --- | --- | --- | --- | --- |
| Total number of patients |  | 194 | 8 | (4.1%) |  |  |  |  |  |  |
| Gender | Male | 87 | 6 | (6.9%) | Reference |  |  | Reference |  |  |
|  | Female | 107 | 2 | (1.9%) | 0.27 | 0.05-1.32 | *p=*0.105 | 0.27 | 0.06-1.36 | *p=*0.113 |
| Age | <Mean | 87 | 5 | (5.7%) | Reference |  |  | Reference |  |  |
|  | ≥Mean | 107 | 3 | (2.8%) | 0.47 | 0.11-1.99 | *p=*0.307 | 0.47 | 0.11-1.96 | *p=*0.298 |
|  | Unknown | 0 | 0 | - |  |  |  |  |  |  |
| Age at onset of PD | <50 | 29 | 3 | (10.3%) | Reference |  |  | Reference |  |  |
|  | ≥50 | 165 | 5 | (3.0%) | 0.28 | 0.07-1.18 | *p=*0.082 | 0.29 | 0.07-1.20 | *p=*0.087 |
|  | Unknown | 0 | 0 | - |  |  |  |  |  |  |
| RBD | Yes | 41 | 1 | (2.4%) | 0.52 | 0.06-4.25 | *p=*0.544 | 0.53 | 0.07-4.30 | *p=*0.551 |
|  | No | 153 | 7 | (4.6%) | Reference |  |  | Reference |  |  |
|  | Not Evaluable | 0 | 0 | - |  |  |  |  |  |  |
|  | Unknown | 0 | 0 | - |  |  |  |  |  |  |
| Daily L-dopa dose | ≤Mean | 105 | 7 | (6.7%) | Reference |  |  | Reference |  |  |
|  | >Mean | 89 | 1 | (1.1%) | 0.16 | 0.02-1.32 | *p=*0.090 | 0.16 | 0.02-1.28 | *p=*0.084 |
|  | Unknown | 0 | 0 | - |  |  |  |  |  |  |
| LED | ≤Mean | 112 | 8 | (7.1%) | Reference |  |  | Reference |  |  |
|  | >Mean | 77 | 0 | (0.0%) | 0.00 | 0.00->999.99 | *p=*0.993 | - | - | - |
|  | Unknown | 5 | 0 | (0.0%) |  |  |  |  |  |  |
| Disease severity （Baseline UPDRS III Score） | ≤Mean | 112 | 3 | (2.7%) | Reference |  |  | Reference |  |  |
|  | >Mean | 82 | 5 | (6.1%) | 2.33 | 0.56-9.74 | *p=*0.247 | 2.31 | 0.55-9.69 | *p=*0.251 |
|  | Unknown | 0 | 0 | - |  |  |  |  |  |  |
| Hyposmia | Yes | 61 | 3 | (4.9%) | 3.40 | 0.35-32.71 | *p=*0.289 | 3.52 | 0.37-33.84 | *p=*0.276 |
|  | No | 68 | 1 | (1.5%) | Reference |  |  | Reference |  |  |
|  | Not Evaluable | 0 | 0 | - |  |  |  |  |  |  |
|  | Unknown | 65 | 4 | (6.2%) |  |  |  |  |  |  |
| Time from the onset of motor symptoms to referral to specialized care (Juntendo University Hospital) | <Mean | 119 | 5 | (4.2%) | Reference |  |  | Reference |  |  |
|  | ≥Mean | 75 | 3 | (4.0%) | 0.96 | 0.23-4.02 | *p=*0.956 | 0.94 | 0.22-3.92 | *p=*0.928 |
|  | Unknown | 0 | 0 | - |  |  |  |  |  |  |
| Orthostatic hypotension | Yes | 44 | 0 | (0.0%) | 0.00 | 0.00->999.99 | *p=*0.995 | 0.00 | 0.00->999.99 | *p=*0.995 |
|  | No | 140 | 7 | (5.0%) | Reference |  |  | Reference |  |  |
|  | Not Evaluable | 1 | 0 | (0.0%) |  |  |  |  |  |  |
|  | Unknown | 9 | 1 | (11.1%) |  |  |  |  |  |  |
| Family history of Parkinson’s disease | First degree | 7 | 1 | (14.3%) | 5.68 | 0.63-50.87 | *p=*0.120 | 5.60 | 0.63-50.15 | *p=*0.123 |
|  | Second degree | 9 | 0 | (0.0%) | 0.00 | 0.00->999.99 | *p=*0.997 | 0.00 | 0.00->999.99 | *p=*0.997 |
|  | Third degree | 9 | 0 | (0.0%) | 0.00 | 0.00->999.99 | *p=*0.997 | 0.00 | 0.00->999.99 | *p=*0.997 |
|  | Fourth degree | 1 | 0 | (0.0%) | 0.00 | 0.00->999.99 | *p=*0.999 | 0.00 | 0.00->999.99 | *p=*0.999 |
|  | None | 142 | 4 | (2.8%) | Reference |  |  | Reference |  |  |
|  | Unknown | 26 | 3 | (11.5%) |  |  |  |  |  |  |
| Constipation | Yes | 111 | 5 | (4.5%) | 1.24 | 0.30-5.20 | *p=*0.766 | 1.20 | 0.29-5.01 | *p=*0.806 |
|  | No | 83 | 3 | (3.6%) | Reference |  |  | Reference |  |  |
|  | Not Evaluable | 0 | 0 | - |  |  |  |  |  |  |
|  | Unknown | 0 | 0 | - |  |  |  |  |  |  |

* adjusted for baseline PD medication use. HR, hazard ratio; CI, confidence interval; RBD, REM sleep behavior disorder; LED, L-dopa equivalent dose; UPDRS, Unified Parkinson’s Disease Rating Scale

Table S6. Cox proportional hazard model(Univariate analysis): Time to UPDRS IV total score two or more

|  |  |  | N (%) | | HR | HR (95%CI) | Wald test | HR adjusted* | HR adjusted (95%CI) | Wald test |
| --- | --- | --- | --- | --- | --- | --- | --- | --- | --- | --- |
| Total number of patients |  | 194 | 136 | (70.1%) |  |  |  |  |  |  |
| Gender | Male | 87 | 59 | (67.8%) | Reference |  |  | Reference |  |  |
|  | Female | 107 | 77 | (72.0%) | 1.15 | 0.82-1.62 | *p=*0.415 | 1.17 | 0.83-1.65 | *p=*0.360 |
| Age | <Mean | 87 | 59 | (67.8%) | Reference |  |  | Reference |  |  |
|  | ≥Mean | 107 | 77 | (72.0%) | 0.99 | 0.70-1.39 | *p=*0.953 | 0.98 | 0.70-1.38 | *p=*0.928 |
|  | Unknown | 0 | 0 | - |  |  |  |  |  |  |
| Age at onset of PD | <50 | 29 | 26 | (89.7%) | Reference |  |  | Reference |  |  |
|  | ≥50 | 165 | 110 | (66.7%) | 0.52 | 0.34-0.80 | *p=*0.003 | 0.53 | 0.34-0.81 | *p=*0.004 |
|  | Unknown | 0 | 0 | - |  |  |  |  |  |  |
| RBD | Yes | 41 | 31 | (75.6%) | 1.37 | 0.91-2.05 | *p=*0.130 | 1.39 | 0.93-2.08 | *p=*0.113 |
|  | No | 153 | 105 | (68.6%) | Reference |  |  | Reference |  |  |
|  | Not Evaluable | 0 | 0 | - |  |  |  |  |  |  |
|  | Unknown | 0 | 0 | - |  |  |  |  |  |  |
| Daily L-dopa dose | ≤Mean | 105 | 61 | (58.1%) | Reference |  |  | Reference |  |  |
|  | >Mean | 89 | 75 | (84.3%) | 2.03 | 1.44-2.86 | *p*<0.001 | 2.01 | 1.43-2.84 | *p*<0.001 |
|  | Unknown | 0 | 0 | - |  |  |  |  |  |  |
| LED | ≤Mean | 112 | 66 | (58.9%) | Reference |  |  | Reference |  |  |
|  | >Mean | 77 | 69 | (89.6%) | 2.66 | 1.87-3.78 | *p*<0.001 | - | - | - |
|  | Unknown | 5 | 1 | (20.0%) |  |  |  |  |  |  |
| Disease severity （Baseline UPDRS III Score） | ≤Mean | 112 | 71 | (63.4%) | Reference |  |  | Reference |  |  |
|  | >Mean | 82 | 65 | (79.3%) | 1.85 | 1.31-2.61 | *p*<0.001 | 1.84 | 1.31-2.60 | *p*<0.001 |
|  | Unknown | 0 | 0 | - |  |  |  |  |  |  |
| Distribution Status of hyposmia | Yes | 61 | 42 | (68.9%) | 1.24 | 0.81-1.90 | *p=*0.323 | 1.26 | 0.82-1.93 | *p=*0.294 |
|  | No | 68 | 43 | (63.2%) | Reference |  |  | Reference |  |  |
|  | Not Evaluable | 0 | 0 | - |  |  |  |  |  |  |
|  | Unknown | 65 | 51 | (78.5%) |  |  |  |  |  |  |
| Time from the onset of motor symptoms to referral to a specialized hospital (Juntendo University) | <Mean | 119 | 66 | (55.5%) | Reference |  |  | Reference |  |  |
|  | ≥Mean | 75 | 70 | (93.3%) | 3.28 | 2.32-4.64 | *p*<0.001 | 3.26 | 2.30-4.61 | *p*<0.001 |
|  | Unknown | 0 | 0 | - |  |  |  |  |  |  |
| Orthostatic hypotension | Yes | 44 | 32 | (72.7%) | 1.17 | 0.78-1.74 | *p=*0.450 | 1.18 | 0.79-1.76 | *p=*0.431 |
|  | No | 140 | 98 | (70.0%) | Reference |  |  | Reference |  |  |
|  | Not Evaluable | 1 | 1 | (100.0%) |  |  |  |  |  |  |
|  | Unknown | 9 | 5 | (55.6%) |  |  |  |  |  |  |
| Family history of Parkinson’s disease | First degree | 7 | 4 | (57.1%) | 0.47 | 0.17-1.29 | *p=*0.144 | 0.47 | 0.17-1.29 | *p=*0.144 |
|  | Second degree | 9 | 7 | (77.8%) | 1.03 | 0.48-2.23 | *p=*0.937 | 1.03 | 0.48-2.23 | *p=*0.941 |
|  | Third degree | 9 | 8 | (88.9%) | 2.17 | 1.04-4.51 | *p=*0.039 | 2.16 | 1.04-4.50 | *p=*0.040 |
|  | Fourth degree | 1 | 0 | (0.0%) | 0.00 | 0.00->999.99 | *p=*0.977 | 0.00 | 0.00->999.99 | *p=*0.977 |
|  | None | 142 | 96 | (67.6%) | Reference |  |  | Reference |  |  |
|  | Unknown | 26 | 21 | (80.8%) |  |  |  |  |  |  |
| Constipation | Yes | 111 | 86 | (77.5%) | 1.83 | 1.28-2.60 | *p*<0.001 | 1.80 | 1.26-2.57 | *p=*0.001 |
|  | No | 83 | 50 | (60.2%) | Reference |  |  | Reference |  |  |
|  | Not Evaluable | 0 | 0 | - |  |  |  |  |  |  |
|  | Unknown | 0 | 0 | - |  |  |  |  |  |  |

* adjusted for baseline PD medication use. HR, hazard ratio; CI, confidence interval; RBD, REM sleep behavior disorder; LED, L-dopa equivalent dose; UPDRS, Unified Parkinson’s Disease Rating Scale

Table S7. Cox proportional hazard model(Univariate analysis): Time to worsening of Hoehn and Yahr severity from baseline

|  |  |  | N (%) | | HR | 95%CI | Wald test | HR adjusted* | 95%CI | Wald test |
| --- | --- | --- | --- | --- | --- | --- | --- | --- | --- | --- |
| Total number of patients |  | 194 | 70 | (36.1%) |  |  |  |  |  |  |
| Gender | Male | 87 | 32 | (36.8%) | Reference |  |  | Reference |  |  |
|  | Female | 107 | 38 | (35.5%) | 0.92 | 0.57-1.47 | *p=*0.727 | 0.95 | 0.59-1.52 | *p=*0.823 |
| Age | <Mean | 87 | 25 | (28.7%) | Reference |  |  | Reference |  |  |
|  | ≥Mean | 107 | 45 | (42.1%) | 1.45 | 0.89-2.36 | *p=*0.139 | 1.43 | 0.87-2.33 | *p=*0.154 |
|  | Unknown | 0 | 0 | - |  |  |  |  |  |  |
| Age at onset of PD | <50 | 29 | 8 | (27.6%) | Reference |  |  | Reference |  |  |
|  | ≧50 | 165 | 62 | (37.6%) | 1.38 | 0.66-2.88 | *p=*0.393 | 1.41 | 0.67-2.94 | *p=*0.364 |
|  | Unknown | 0 | 0 | - |  |  |  |  |  |  |
| RBD | Yes | 41 | 13 | (31.7%) | 0.80 | 0.44-1.45 | *p=*0.458 | 0.81 | 0.44-1.47 | *p=*0.483 |
|  | No | 153 | 57 | (37.3%) | Reference |  |  | Reference |  |  |
|  | Not Evaluable | 0 | 0 | - |  |  |  |  |  |  |
|  | Unknown | 0 | 0 | - |  |  |  |  |  |  |
| Daily L-dopa dose | ≤Mean | 105 | 46 | (43.8%) | Reference |  |  | Reference |  |  |
|  | >Mean | 89 | 24 | (27.0%) | 0.51 | 0.31-0.84 | *p=*0.008 | 0.49 | 0.30-0.81 | *p=*0.005 |
|  | Unknown | 0 | 0 | - |  |  |  |  |  |  |
| LED | ≤Mean | 112 | 48 | (42.9%) | Reference |  |  | Reference |  |  |
|  | >Mean | 77 | 22 | (28.6%) | 0.62 | 0.37-1.02 | *p=*0.061 | - | - | - |
|  | Unknown | 5 | 0 | (0.0%) |  |  |  |  |  |  |
| Disease severity （Baseline UPDRS III Score） | ≤Mean | 112 | 50 | (44.6%) | Reference |  |  | Reference |  |  |
|  | >Mean | 82 | 20 | (24.4%) | 0.46 | 0.28-0.78 | *p=*0.004 | 0.46 | 0.27-0.77 | *p=*0.003 |
|  | Unknown | 0 | 0 | - |  |  |  |  |  |  |
| Hyposmia | Yes | 61 | 21 | (34.4%) | 1.05 | 0.58-1.89 | *p=*0.877 | 1.09 | 0.60-1.97 | *p=*0.771 |
|  | No | 68 | 23 | (33.8%) | Reference |  |  | Reference |  |  |
|  | Not Evaluable | 0 | 0 | - |  |  |  |  |  |  |
|  | Unknown | 65 | 26 | (40.0%) |  |  |  |  |  |  |
| Distribution of time from the onset of motor symptoms to referral to specialized care (Juntendo University Hospital) | <Mean | 119 | 51 | (42.9%) | Reference |  |  | Reference |  |  |
|  | ≥Mean | 75 | 19 | (25.3%) | 0.50 | 0.29-0.84 | *p=*0.010 | 0.48 | 0.28-0.82 | *p=*0.007 |
|  | Unknown | 0 | 0 | - |  |  |  |  |  |  |
| Orthostatic hypotension | Yes | 44 | 12 | (27.3%) | 0.64 | 0.34-1.20 | *p=*0.163 | 0.65 | 0.35-1.22 | *p=*0.184 |
|  | No | 140 | 55 | (39.3%) | Reference |  |  | Reference |  |  |
|  | Not Evaluable | 1 | 0 | (0.0%) |  |  |  |  |  |  |
|  | Unknown | 9 | 3 | (33.3%) |  |  |  |  |  |  |
| Family history of Parkinson’ disease | First degree | 7 | 3 | (42.9%) | 1.37 | 0.43-4.39 | *p=*0.597 | 1.35 | 0.42-4.32 | *p=*0.614 |
|  | Second degree | 9 | 4 | (44.4%) | 1.20 | 0.43-3.32 | *p=*0.727 | 1.18 | 0.43-3.27 | *p=*0.748 |
|  | Third degree | 9 | 2 | (22.2%) | 0.58 | 0.14-2.38 | *p=*0.448 | 0.57 | 0.14-2.34 | *p=*0.436 |
|  | Fourth degree | 1 | 1 | (100.0%) | 5.26 | 0.71-38.70 | *p=*0.103 | 5.18 | 0.70-38.11 | *p=*0.106 |
|  | None | 142 | 52 | (36.6%) | Reference |  |  | Reference |  |  |
|  | Unknown | 26 | 8 | (30.8%) |  |  |  |  |  |  |
| Constipation | Yes | 111 | 43 | (38.7%) | 1.25 | 0.78-2.03 | *p=*0.356 | 1.21 | 0.75-1.95 | *p=*0.442 |
|  | No | 83 | 27 | (32.5%) | Reference |  |  | Reference |  |  |
|  | Not Evaluable | 0 | 0 | - |  |  |  |  |  |  |
|  | Unknown | 0 | 0 | - |  |  |  |  |  |  |

* adjusted for baseline PD medication use. HR, hazard ratio; CI, confidence interval; RBD, REM sleep behavior disorder; LED, L-dopa equivalent dose; UPDRS, Unified Parkinson’s Disease Rating Scale

Table S8. Cox proportional hazard model (Multivariate analysis): Time to worsening of PD symptom defined as doubling of LED from baseline

|  |  |  | N (%) | | HR adjusted* | 95%CI | Wald test |
| --- | --- | --- | --- | --- | --- | --- | --- |
| Total number of patients |  | 194 | 15 | (7.7%) |  |  |  |
| Gender | Male | 87 | 11 | (12.6%) | Reference |  |  |
|  | Female | 107 | 4 | (3.7%) | 0.44 | 0.13-1.51 | *p=*0.191 |
| Daily L-dopa dose | ≤Mean | 105 | 13 | (12.4%) | Reference |  |  |
|  | >Mean | 89 | 2 | (2.2%) | 0.12 | 0.02-0.95 | *p=*0.044 |
|  | Unknown | 0 | 0 | - |  |  |  |
| Family history of Parkinson’s disease | Yes | 25 | 3 | (12.0%) | 2.39 | 0.63-9.01 | *p=*0.200 |
|  | No | 143 | 8 | (5.6%) | Reference |  |  |
|  | Unknown | 26 | 4 | (15.4%) |  |  |  |

* adjusted for baseline PD medication use. HR, hazard ratio; CI, confidence interval

Table S9. Cox proportional hazard model (Multivariate analysis): Time to UPDRS IV total score two or more

|  |  |  | N (%) | | HR adjusted* | 95%CI | Wald test |
| --- | --- | --- | --- | --- | --- | --- | --- |
| Total number of patients |  | 194 | 136 | (70.1%) |  |  |  |
| Age at onset of PD | <50 | 29 | 26 | (89.7%) | Reference |  |  |
|  | ≥50 | 165 | 110 | (66.7%) | 0.69 | 0.43-1.12 | *p=*0.136 |
|  | Unknown | 0 | 0 | - |  |  |  |
| Daily L-dopa dose | ≤Mean | 105 | 61 | (58.1%) | Reference |  |  |
|  | >Mean | 89 | 75 | (84.3%) | 1.17 | 0.75-1.82 | *p=*0.502 |
|  | Unknown | 0 | 0 | - |  |  |  |
| Disease severity （Baseline UPDRS III Score） | ≤Mean | 112 | 71 | (63.4%) | Reference |  |  |
|  | >Mean | 82 | 65 | (79.3%) | 1.29 | 0.83-2.00 | *p=*0.267 |
|  | Unknown | 0 | 0 | - |  |  |  |
| Time from the onset of motor symptoms to referral to specialized care (Juntendo University Hospital) | <Mean | 119 | 66 | (55.5%) | Reference |  |  |
|  | ≥Mean | 75 | 70 | (93.3%) | 2.69 | 1.73-4.19 | *p*<0.001 |
|  | Unknown | 0 | 0 | - |  |  |  |
| Family history of Parkinson’s disease | Yes | 25 | 19 | (76.0%) | 1.08 | 0.65-1.79 | *p=*0.770 |
|  | No | 143 | 96 | (67.1%) | Reference |  |  |
|  | Unknown | 26 | 21 | (80.8%) |  |  |  |
| Constipation | Yes | 111 | 86 | (77.5%) | 1.39 | 0.93-2.09 | *p=*0.111 |
|  | No | 83 | 50 | (60.2%) | Reference |  |  |
|  | Not Evaluable | 0 | 0 | - |  |  |  |
|  | Unknown | 0 | 0 | - |  |  |  |

* adjusted for baseline PD medication use. HR, hazard ratio; CI, confidence interval; UPDRS, Unified Parkinson’s Disease Rating Scale

Table S10. Cox proportional hazard model (Multivariate analysis): Time to worsening of Hoehn and Yahr severity from baseline

|  |  |  | N (%) | | HR adjusted* | 95%CI | Wald test |
| --- | --- | --- | --- | --- | --- | --- | --- |
| Total number of patients |  | 194 | 70 | (36.1%) |  |  |  |
| Daily L-dopa dose | ≤Mean | 105 | 46 | (43.8%) | Reference |  |  |
|  | >Mean | 89 | 24 | (27.0%) | 0.71 | 0.40-1.25 | *p=*0.234 |
|  | Unknown | 0 | 0 | - |  |  |  |
| Disease severity （Baseline UPDRS III Score） | ≤Mean | 112 | 50 | (44.6%) | Reference |  |  |
|  | >Mean | 82 | 20 | (24.4%) | 0.61 | 0.34-1.10 | *p=*0.099 |
|  | Unknown | 0 | 0 | - |  |  |  |
| Time from the onset of motor symptoms to referral to specialized care (Juntendo University Hospital) | <Mean | 119 | 51 | (42.9%) | Reference |  |  |
|  | ≥Mean | 75 | 19 | (25.3%) | 0.65 | 0.37-1.16 | *p=*0.149 |
|  | Unknown | 0 | 0 | - |  |  |  |

* adjusted for baseline PD medication use. HR, hazard ratio; CI, confidence interval; UPDRS, Unified Parkinson’s Disease Rating Scale

Table S11. Logistic regression model (Univariate analysis): UPDRS III total score worsened by 25% from baseline at 24 months

|  |  |  | N (%) | | OR | 95%CI | Wald test | Adjusted OR* | 95%CI | Wald test |
| --- | --- | --- | --- | --- | --- | --- | --- | --- | --- | --- |
| Total number of patients |  | 163 | 41 | (25.2%) |  |  |  |  |  |  |
| Gender | Male | 71 | 14 | (19.7%) | Reference |  |  | Reference |  |  |
|  | Female | 92 | 27 | (29.3%) | 1.69 | 0.81-3.53 | *p=*0.162 | 1.57 | 0.74-3.30 | *p=*0.238 |
| Age | <Mean | 70 | 17 | (24.3%) | Reference |  |  | Reference |  |  |
|  | ≥Mean | 93 | 24 | (25.8%) | 1.08 | 0.53-2.22 | *p=*0.825 | 1.10 | 0.53-2.29 | *p=*0.791 |
|  | Unknown | 0 | 0 | - |  |  |  |  |  |  |
| Age at onset of PD | <50 | 26 | 6 | (23.1%) | Reference |  |  | Reference |  |  |
|  | ≥50 | 137 | 35 | (25.5%) | 1.14 | 0.43-3.08 | *p=*0.790 | 1.08 | 0.40-2.91 | *p=*0.882 |
|  | Unknown | 0 | 0 | - |  |  |  |  |  |  |
| RBD | Yes | 34 | 11 | (32.4%) | 1.58 | 0.69-3.61 | *p=*0.279 | 1.48 | 0.63-3.47 | *p=*0.362 |
|  | No | 129 | 30 | (23.3%) | Reference |  |  | Reference |  |  |
|  | Not Evaluable | 0 | 0 | - |  |  |  |  |  |  |
|  | Unknown | 0 | 0 | - |  |  |  |  |  |  |
| Daily L-dopa dose | ≤Mean | 83 | 26 | (31.3%) | Reference |  |  | Reference |  |  |
|  | >Mean | 80 | 15 | (18.8%) | 0.51 | 0.24-1.05 | *p=*0.067 | 0.55 | 0.26-1.14 | *p=*0.110 |
|  | Unknown | 0 | 0 | - |  |  |  |  |  |  |
| LED | <=Mean | 93 | 25 | (26.9%) | Reference |  |  | Reference |  |  |
|  | >Mean | 67 | 13 | (19.4%) | 0.65 | 0.31-1.40 | *p=*0.275 | - | - | - |
|  | Unknown | 3 | 3 | (100.0%) |  |  |  |  |  |  |
| Disease severity （Baseline UPDRS III Score） | ≤Mean | 93 | 37 | (39.8%) | Reference |  |  | Reference |  |  |
|  | >Mean | 70 | 4 | (5.7%) | 0.09 | 0.03-0.27 | *p*<0.001 | 0.10 | 0.03-0.29 | *p*<0.001 |
|  | Unknown | 0 | 0 | - |  |  |  |  |  |  |
| Hyposmia | Yes | 52 | 12 | (23.1%) | 1.08 | 0.43-2.67 | *p=*0.876 | 0.90 | 0.35-2.30 | *p=*0.819 |
|  | No | 55 | 12 | (21.8%) | Reference |  |  | Reference |  |  |
|  | Not Evaluable | 0 | 0 | - |  |  |  |  |  |  |
|  | Unknown | 56 | 17 | (30.4%) |  |  |  |  |  |  |
| Time from the onset of motor symptoms to referral to a specialized hospital (Juntendo University) | <Mean | 93 | 25 | (26.9%) | Reference |  |  | Reference |  |  |
|  | ≥Mean | 70 | 16 | (22.9%) | 0.81 | 0.39-1.66 | *p=*0.558 | 0.88 | 0.42-1.82 | *p=*0.723 |
|  | Unknown | 0 | 0 | - |  |  |  |  |  |  |
| Orthostatic hypotension | Yes | 33 | 14 | (42.4%) | 2.75 | 1.22-6.21 | *p=*0.015 | 2.65 | 1.16-6.10 | *p=*0.021 |
|  | No | 123 | 26 | (21.1%) | Reference |  |  | Reference |  |  |
|  | Not Evaluable | 1 | 0 | (0.0%) |  |  |  |  |  |  |
|  | Unknown | 6 | 1 | (16.7%) |  |  |  |  |  |  |
| Family history of Parkinson’s disease | First degree | 7 | 3 | (42.9%) | 2.25 | 0.48-10.65 | *p=*0.964 | 2.33 | 0.49-11.05 | *p=*0.964 |
|  | Second degree | 8 | 2 | (25.0%) | 1.00 | 0.19-5.23 | *p=*0.974 | 1.04 | 0.20-5.43 | *p=*0.974 |
|  | Third degree | 8 | 1 | (12.5%) | 0.43 | 0.05-3.63 | *p=*0.985 | 0.44 | 0.05-3.77 | *p=*0.985 |
|  | Fourth degree | 1 | 0 | (0.0%) | <0.01 | <0.01->999.99 | *p=*0.974 | <0.01 | <0.01->999.99 | *p=*0.974 |
|  | None | 116 | 29 | (25.0%) | Reference |  |  | Reference |  |  |
|  | Unknown | 23 | 6 | (26.1%) |  |  |  |  |  |  |
| Constipation | Yes | 92 | 20 | (21.7%) | 0.66 | 0.32-1.35 | *p=*0.254 | 0.73 | 0.35-1.51 | *p=*0.396 |
|  | No | 71 | 21 | (29.6%) | Reference |  |  | Reference |  |  |
|  | Not Evaluable | 0 | 0 | - |  |  |  |  |  |  |
|  | Unknown | 0 | 0 | - |  |  |  |  |  |  |

* adjusted for baseline PD medication use. OR, odds ratio; CI, confidence interval; RBD, REM sleep behavior disorder; LED, L-dopa equivalent dose; UPDRS, Unified Parkinson’s Disease Rating Scale

Table S12. Logistic regression model (Univariate analysis): UPDRS III total score improved or maintained from baseline to 24 months

|  |  |  | N (%) | | OR | 95%CI | Wald test | Adjusted OR* | 95%CI | Wald test |
| --- | --- | --- | --- | --- | --- | --- | --- | --- | --- | --- |
| Total number of patients |  | 169 | 57 | (33.7%) |  |  |  |  |  |  |
| Gender | Male | 74 | 24 | (32.4%) | Reference |  |  | Reference |  |  |
|  | Female | 95 | 33 | (34.7%) | 1.11 | 0.58-2.11 | *p=*0.753 | 1.17 | 0.61-2.23 | *p=*0.643 |
| Age | <Mean | 74 | 29 | (39.2%) | Reference |  |  | Reference |  |  |
|  | ≥Mean | 95 | 28 | (29.5%) | 0.65 | 0.34-1.23 | *p=*0.186 | 0.63 | 0.33-1.20 | *p=*0.160 |
|  | Unknown | 0 | 0 | - |  |  |  |  |  |  |
| Age at onset of PD | <50 | 26 | 8 | (30.8%) | Reference |  |  | Reference |  |  |
|  | ≥50 | 143 | 49 | (34.3%) | 1.17 | 0.48-2.89 | *p=*0.729 | 1.21 | 0.49-2.99 | *p=*0.677 |
|  | Unknown | 0 | 0 | - |  |  |  |  |  |  |
| RBD | Yes | 35 | 13 | (37.1%) | 1.21 | 0.56-2.62 | *p=*0.632 | 1.24 | 0.57-2.70 | *p=*0.592 |
|  | No | 134 | 44 | (32.8%) | Reference |  |  | Reference |  |  |
|  | Not Evaluable | 0 | 0 | - |  |  |  |  |  |  |
|  | Unknown | 0 | 0 | - |  |  |  |  |  |  |
| Daily L-dopa dose | ≤Mean | 86 | 30 | (34.9%) | Reference |  |  | Reference |  |  |
|  | >Mean | 83 | 27 | (32.5%) | 0.90 | 0.48-1.70 | *p=*0.746 | 0.85 | 0.45-1.62 | *p=*0.624 |
|  | Unknown | 0 | 0 | - |  |  |  |  |  |  |
| LED | ≤Mean | 94 | 33 | (35.1%) | Reference |  |  | Reference |  |  |
|  | >Mean | 70 | 24 | (34.3%) | 0.96 | 0.50-1.85 | *p=*0.913 | - | - | - |
|  | Unknown | 5 | 0 | (0.0%) |  |  |  |  |  |  |
| Disease severity （Baseline UPDRS III Score） | ≤Mean | 96 | 21 | (21.9%) | Reference |  |  | Reference |  |  |
|  | >Mean | 73 | 36 | (49.3%) | 3.47 | 1.78-6.77 | *p*<0.001 | 3.48 | 1.78-6.79 | *p*<0.001 |
|  | Unknown | 0 | 0 | - |  |  |  |  |  |  |
| Hyposmia | Yes | 53 | 21 | (39.6%) | 0.82 | 0.38-1.77 | *p=*0.614 | 0.88 | 0.40-1.90 | *p=*0.735 |
|  | No | 54 | 24 | (44.4%) | Reference |  |  | Reference |  |  |
|  | Not Evaluable | 0 | 0 | - |  |  |  |  |  |  |
|  | Unknown | 62 | 12 | (19.4%) |  |  |  |  |  |  |
| Time from the onset of motor symptoms to referral to specialized care (Juntendo University Hospital) | <Mean | 98 | 36 | (36.7%) | Reference |  |  | Reference |  |  |
|  | ≥Mean | 71 | 21 | (29.6%) | 0.72 | 0.38-1.39 | *p=*0.332 | 0.69 | 0.36-1.33 | *p=*0.265 |
|  | Unknown | 0 | 0 | - |  |  |  |  |  |  |
| Orthostatic hypotension | Yes | 37 | 10 | (27.0%) | 0.63 | 0.28-1.41 | *p=*0.262 | 0.64 | 0.28-1.46 | *p=*0.290 |
|  | No | 124 | 46 | (37.1%) | Reference |  |  | Reference |  |  |
|  | Not Evaluable | 1 | 1 | (100.0%) |  |  |  |  |  |  |
|  | Unknown | 7 | 0 | (0.0%) |  |  |  |  |  |  |
| Family history of Parkinson’s disease | First degree | 7 | 3 | (42.9%) | 1.79 | 0.38-8.41 | *p=*0.968 | 1.75 | 0.37-8.22 | *p=*0.968 |
|  | Second degree | 8 | 3 | (37.5%) | 1.43 | 0.33-6.32 | *p=*0.965 | 1.40 | 0.32-6.17 | *p=*0.965 |
|  | Third degree | 8 | 6 | (75.0%) | 7.17 | 1.38-37.20 | *p=*0.986 | 7.00 | 1.35-36.35 | *p=*0.986 |
|  | Fourth degree | 1 | 1 | (100.0%) | >999.99 | <0.01->999.99 | *p=*0.970 | >999.99 | <0.01->999.99 | *p=*0.970 |
|  | None | 122 | 36 | (29.5%) | Reference |  |  | Reference |  |  |
|  | Unknown | 23 | 8 | (34.8%) |  |  |  |  |  |  |
| Constipation | Yes | 98 | 34 | (34.7%) | 1.11 | 0.58-2.12 | *p=*0.755 | 1.04 | 0.54-2.00 | *p=*0.908 |
|  | No | 71 | 23 | (32.4%) | Reference |  |  | Reference |  |  |
|  | Not Evaluable | 0 | 0 | - |  |  |  |  |  |  |
|  | Unknown | 0 | 0 | - |  |  |  |  |  |  |

* adjusted for baseline PD medication use. OR, odds ratio; CI, confidence interval; RBD, REM sleep behavior disorder; LED, L-dopa equivalent dose; UPDRS, Unified Parkinson’s Disease Rating Scale

Table S13. Logistic regression model(Univariate analysis): Hoehn and Yahr severity increased 1 point or more from baseline at 24 months

|  |  |  | N (%) | | OR | 95%CI | Wald test | Adjusted OR* | 95%CI | Wald test |
| --- | --- | --- | --- | --- | --- | --- | --- | --- | --- | --- |
| Total number of patients |  | 120 | 55 | (45.8%) |  |  |  |  |  |  |
| Gender | Male | 49 | 28 | (57.1%) | Reference |  |  | Reference |  |  |
|  | Female | 71 | 27 | (38.0%) | 0.46 | 0.22-0.97 | *p=*0.040 | 0.48 | 0.23-1.01 | *p=*0.055 |
| Age | <Mean | 46 | 19 | (41.3%) | Reference |  |  | Reference |  |  |
|  | ≥Mean | 74 | 36 | (48.6%) | 1.35 | 0.64-2.83 | *p=*0.433 | 1.33 | 0.63-2.81 | *p=*0.454 |
|  | Unknown | 0 | 0 | - |  |  |  |  |  |  |
| Age at onset of PD | <50 | 15 | 6 | (40.0%) | Reference |  |  | Reference |  |  |
|  | ≥50 | 105 | 49 | (46.7%) | 1.31 | 0.44-3.95 | *p=*0.629 | 1.36 | 0.45-4.10 | *p=*0.584 |
|  | Unknown | 0 | 0 | - |  |  |  |  |  |  |
| RBD | Yes | 33 | 12 | (36.4%) | 0.58 | 0.26-1.33 | *p=*0.202 | 0.60 | 0.26-1.38 | *p=*0.228 |
|  | No | 87 | 43 | (49.4%) | Reference |  |  | Reference |  |  |
|  | Not Evaluable | 0 | 0 | - |  |  |  |  |  |  |
|  | Unknown | 0 | 0 | - |  |  |  |  |  |  |
| Daily L-dopa dose | ≤Mean | 56 | 34 | (60.7%) | Reference |  |  | Reference |  |  |
|  | >Mean | 64 | 21 | (32.8%) | 0.32 | 0.15-0.67 | *p=*0.003 | 0.29 | 0.13-0.61 | *p=*0.001 |
|  | Unknown | 0 | 0 | - |  |  |  |  |  |  |
| LED | ≤Mean | 60 | 36 | (60.0%) | Reference |  |  | Reference |  |  |
|  | >Mean | 58 | 19 | (32.8%) | 0.32 | 0.15-0.69 | *p=*0.003 | - | - | - |
|  | Unknown | 2 | 0 | (0.0%) |  |  |  |  |  |  |
| Disease severity （Baseline UPDRS III Score） | ≤Mean | 65 | 40 | (61.5%) | Reference |  |  | Reference |  |  |
|  | >Mean | 55 | 15 | (27.3%) | 0.23 | 0.11-0.51 | *p*<0.001 | 0.22 | 0.10-0.47 | *p*<0.001 |
|  | Unknown | 0 | 0 | - |  |  |  |  |  |  |
| Hyposmia | Yes | 39 | 17 | (43.6%) | 0.77 | 0.32-1.90 | *p=*0.573 | 0.85 | 0.34-2.11 | *p=*0.725 |
|  | No | 38 | 19 | (50.0%) | Reference |  |  | Reference |  |  |
|  | Not Evaluable | 0 | 0 | - |  |  |  |  |  |  |
|  | Unknown | 43 | 19 | (44.2%) |  |  |  |  |  |  |
| Time from the onset of motor symptoms to referral to a specialized hospital (Juntendo University) | <Mean | 61 | 39 | (63.9%) | Reference |  |  | Reference |  |  |
|  | ≥Mean | 59 | 16 | (27.1%) | 0.21 | 0.10-0.46 | *p*<0.001 | 0.19 | 0.09-0.42 | *p*<0.001 |
|  | Unknown | 0 | 0 | - |  |  |  |  |  |  |
| Orthostatic hypotension | Yes | 27 | 8 | (29.6%) | 0.40 | 0.16-1.02 | *p=*0.054 | 0.41 | 0.16-1.05 | *p=*0.065 |
|  | No | 88 | 45 | (51.1%) | Reference |  |  | Reference |  |  |
|  | Not Evaluable | 0 | 0 | - |  |  |  |  |  |  |
|  | Unknown | 5 | 2 | (40.0%) |  |  |  |  |  |  |
| Family history of Parkinson’s disease | First degree | 6 | 3 | (50.0%) | 1.05 | 0.20-5.54 | *p=*0.968 | 1.03 | 0.19-5.41 | *p=*0.968 |
|  | Second degree | 6 | 4 | (66.7%) | 2.11 | 0.36-12.17 | *p=*0.978 | 2.05 | 0.35-11.87 | *p=*0.978 |
|  | Third degree | 7 | 2 | (28.6%) | 0.42 | 0.08-2.30 | *p=*0.954 | 0.41 | 0.08-2.25 | *p=*0.954 |
|  | Fourth degree | 1 | 1 | (100.0%) | >999.99 | <0.01->999.99 | *p=*0.967 | >999.99 | <0.01->999.99 | *p=*0.967 |
|  | None | 78 | 38 | (48.7%) | Reference |  |  | Reference |  |  |
|  | Unknown | 22 | 7 | (31.8%) |  |  |  |  |  |  |
| Constipation | Yes | 75 | 35 | (46.7%) | 1.09 | 0.52-2.30 | *p=*0.813 | 1.01 | 0.47-2.13 | *p=*0.987 |
|  | No | 45 | 20 | (44.4%) | Reference |  |  | Reference |  |  |
|  | Not Evaluable | 0 | 0 | - |  |  |  |  |  |  |
|  | Unknown | 0 | 0 | - |  |  |  |  |  |  |

* adjusted for baseline PD medication use. OR, odds ratio; CI, confidence interval; RBD, REM sleep behavior disorder; LED, L-dopa equivalent dose; UPDRS, Unified Parkinson’s Disease Rating Scale

Table S14. Logistic regression model (Multivariate analysis): UPDRS III total score worsened by 25% from baseline at 24 months

|  |  |  | N (%) | | OR adjusted* | 95%CI | Wald test |
| --- | --- | --- | --- | --- | --- | --- | --- |
| Total number of patients |  | 163 | 41 | (25.2%) |  |  |  |
| Disease severity （Baseline UPDRS III Score） | <=Mean | 93 | 37 | (39.8%) | Reference |  |  |
|  | >Mean | 70 | 4 | (5.7%) | 0.08 | 0.03-0.27 | *p*<0.001 |
|  | Unknown | 0 | 0 | - |  |  |  |
| Orthostatic hypotension | Yes | 33 | 14 | (42.4%) | 3.81 | 1.43-10.13 | *p=*0.007 |
|  | No | 123 | 26 | (21.1%) | Reference |  |  |
|  | Not Evaluable | 1 | 0 | (0.0%) |  |  |  |
|  | Unknown | 6 | 1 | (16.7%) |  |  |  |

* adjusted for baseline PD medication use. OR, odds ratio; CI, confidence interval; UPDRS, Unified Parkinson’s Disease Rating Scale

Table S15. Logistic regression model (Multivariate analysis): Hoehn and Yahr severity increased 1 point or more from baseline at 24 months

|  |  |  | N (%) | | OR adjusted | 95%CI | Wald test |
| --- | --- | --- | --- | --- | --- | --- | --- |
| Total number of patients |  | 120 | 55 | (45.8%) |  |  |  |
| Daily L-dopa dose | ≤Mean | 56 | 34 | (60.7%) | Reference |  |  |
|  | >Mean | 64 | 21 | (32.8%) | 0.55 | 0.23-1.31 | *p=*0.176 |
|  | Unknown | 0 | 0 | - |  |  |  |
| Disease severity （Baseline UPDRS III Score） | ≤Mean | 65 | 40 | (61.5%) | Reference |  |  |
|  | >Mean | 55 | 15 | (27.3%) | 0.36 | 0.15-0.87 | *p=*0.023 |
|  | Unknown | 0 | 0 | - |  |  |  |
| Time from the onset of motor symptoms to referral to specialized care (Juntendo University Hospital) | <Mean | 61 | 39 | (63.9%) | Reference |  |  |
|  | ≥Mean | 59 | 16 | (27.1%) | 0.29 | 0.13-0.69 | *p=*0.005 |
|  | Unknown | 0 | 0 | - |  |  |  |

* adjusted for baseline PD medication use. OR, odds ratio; CI, confidence interval; UPDRS, Unified Parkinson’s Disease Rating Scale

Table S16. Baseline demographics and clinical characteristics (Analysis set vs whole population)

| Subject Characteristics | Analysis set (N = 194) | Whole population (N = 337) |
| --- | --- | --- |
| Sex |  |  |
| Male, *n* (%) | 87 (44.8) | 150 (44.5) |
| Age at onset of PD |  |  |
| Mean (SD) | 60.9 (11.0) | 61.7 (11.0) |
| Age at entry |  |  |
| Mean (SD) | 66.4 (10.1) | 66.7 (10.5) |
| Disease duration (year) |  |  |
| Mean (SD) | 6.22 (5.25) | 5.65 (4.86) |
| Family history of PD, *n* (%) |  |  |
| First degree | 7 (3.6) | 12 (3.6) |
| Second degree | 9 (4.6) | 11 (3.3) |
| Third degree | 9 (4.6) | 10 (3.0) |
| Forth degree | 1 (0.5) | 2 (0.6) |
| None | 142 (73.2) | 198 (58.8) |
| Unknown | 26 (13.4) | 104 (30.9) |
| Time from onset of motor symptoms to referral to specialized care (Juntendo University Hospital) |  |  |
| Mean (SD) | 2359.4 (1874.7) | 2184.56 (1714.00) |
| Hoehn and Yahr Score |  |  |
| 1 *n* (%) | 47 (24.2) | 75 (25.3) |
| 2 *n* (%) | 61 (31.4) | 116 (39.1) |
| 3 *n* (%) | 55 (28.4) | 85 (28.6) |
| 4 *n* (%) | 9 (4.6) | 16 (5.4) |
| 5 *n* (%) | 3 (1.5) | 5 (1.7) |
| Mean (SD) | 2.2 (1.0)^a^ | 2.2 (0.93)^c^ |
| UPDRS III Total Score |  |  |
| Mean (SD) | 20.0 (12.6) | 19.6 (12.45) |
| Daily L-dopa equivalent dose |  |  |
| Mean (SD) | 616.6 (364.8)^b^ | 583.9 (376.9) |

a; N = 174, b; N = 189, c; N = 297


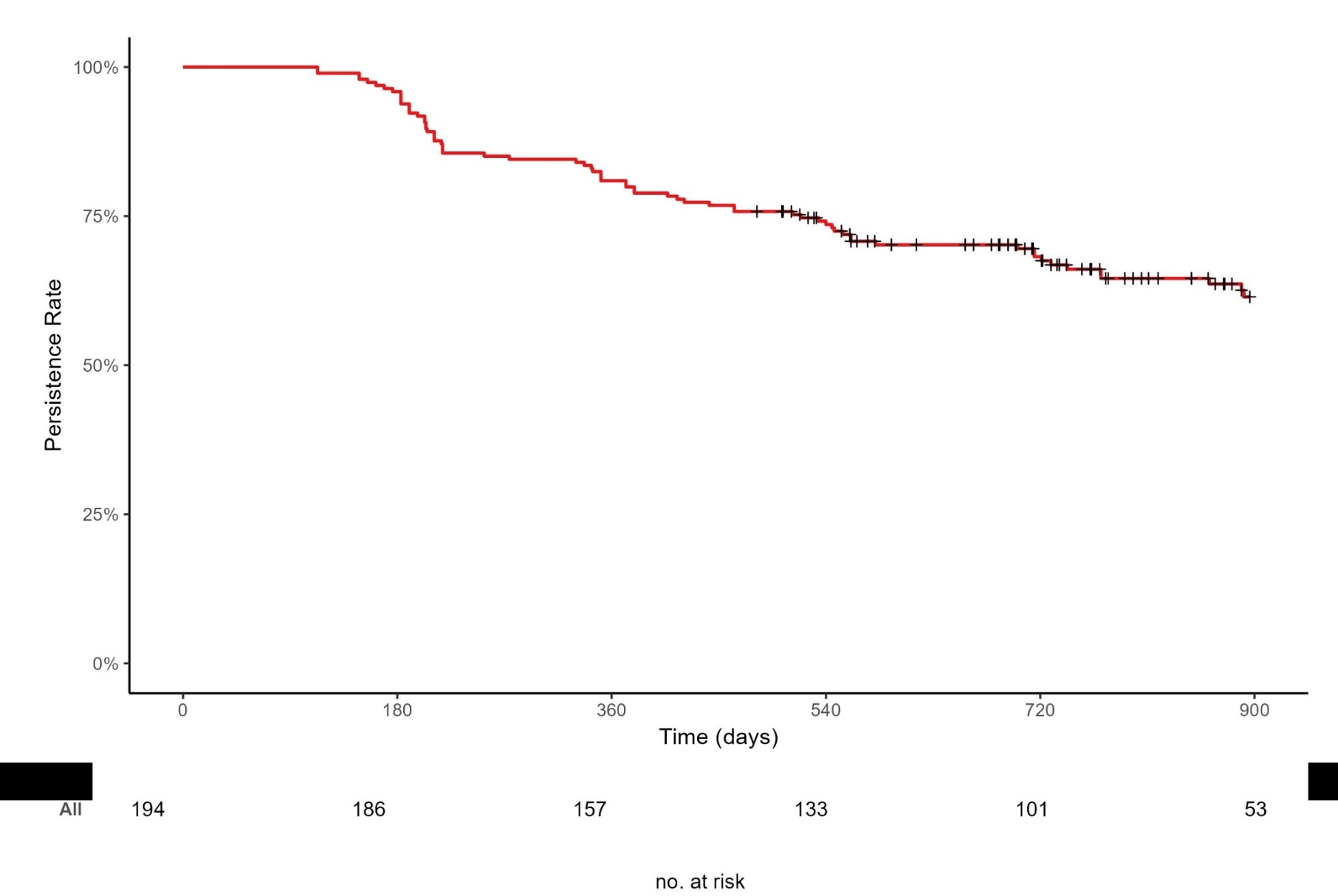


Figure S1. Kaplan-Meier curve of freedom from clinically relevant worsening, defined as worsening of UPDRS III score by 5 points


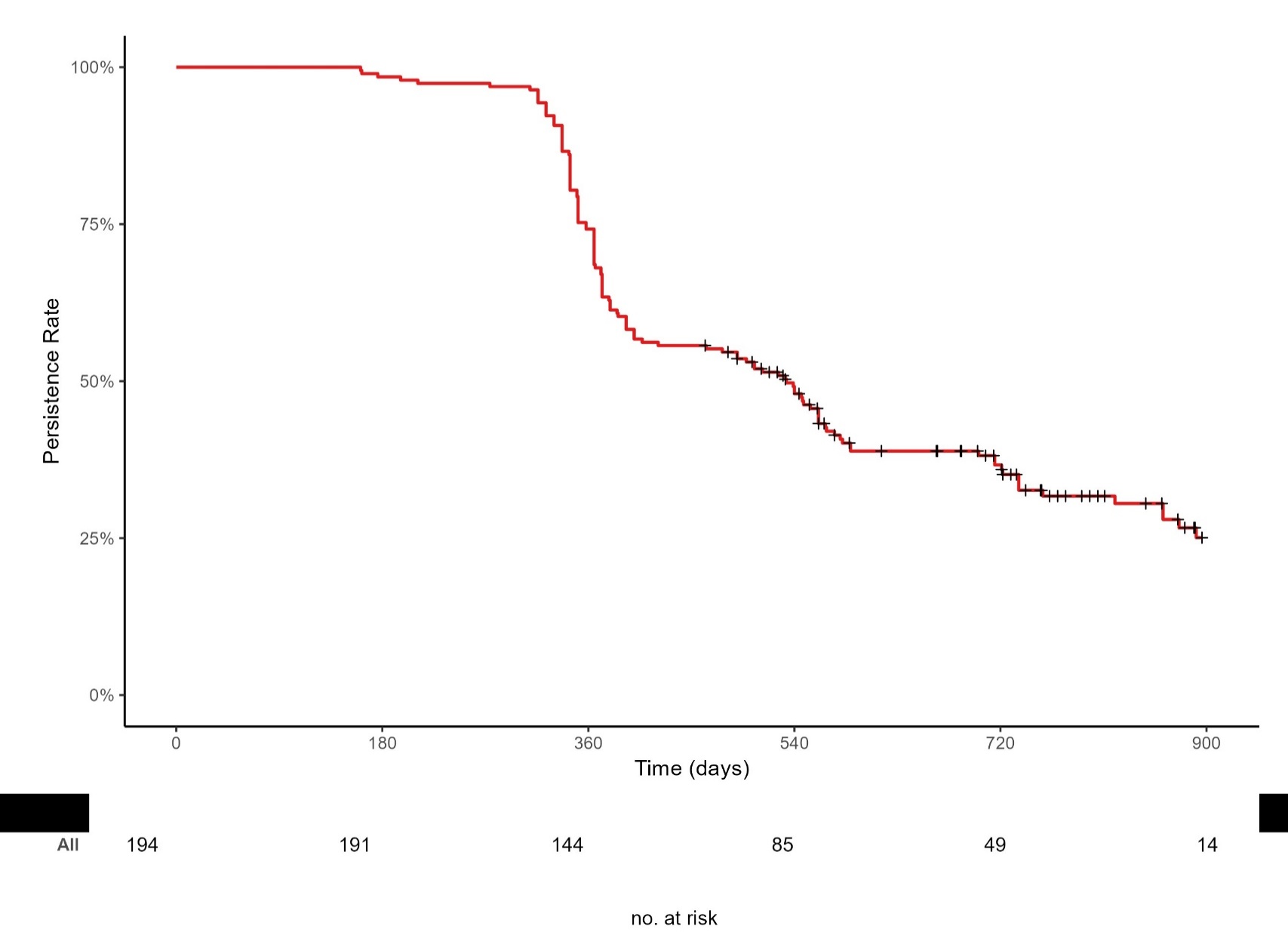


Figure S2. Kaplan-Meier curve of freedom from clinically relevant worsening, defined as UPDRS IV score reaching 2 points

**Supplement 2 Exploration of rapid progressors**

**Method:**

In this study, rapid progressor was defined as subgroup with top 10 percentile of annual UPDRS III score worsening, ie, worsening of UPDRS III score divided by observation period. To explore the factors associated with rapid disease progression, baseline characteristics were listed, and were compared with those for patients exhibited stable disease or improvement during observation period. For rapid progressors, PD related mutations were analyzed using Ion torrent AmpliSeq panel which included 37 PD or dementia associated genes (Table S16) [1].

**Results:**

Baseline characteristics of rapid progressors are listed in Table S17. Baseline characteristics were compared between early progressors and patients exhibited stable disease or improvement, and none of them was statistically significant (Table S18). No genetic mutation was identified from early progressors, except for one with mutation in GBA gene identified.

**Conclusion:**

From this exploration, no trend was seen in terms of baseline characteristics of rapid progressors.

Table S17. Genes analyzed [1]

| **Genes related to PD** | **Genes related to dementia** |
| --- | --- |
| SNCA(PARK1,4) | MAPT |
| parkin(PARK2) | PSEN1 |
| UCH-L1(PARK5) | GRN |
| PINK1(PARK6) | APP |
| DJ-1(PARK7) | APOE |
| LRRK2(PARK8) |  |
| ATP13A2(PARK9) |  |
| GIGYF2(PARK11) |  |
| HTRA2(PARK13) |  |
| PLA2G6(PARK14) |  |
| FBXO7(PARK15) |  |
| VPS35 (PARK17) |  |
| EIF4G1 (PARKk18) |  |
| DNAJC6(PaRK19) |  |
| SYNJ1(PARK20) |  |
| DNAJC13(PARK21) |  |
| CHCHD2(PARK22) |  |
| VPS13C(PARK23) |  |
| GCH1 |  |
| NR4A2 |  |
| RAB7L1 |  |
| BST1 |  |
| C19orf12 |  |
| RAB39B |  |
| VPS13D |  |
| LRP10 |  |
| VPS13B |  |
| VPS13A |  |
| UQCRC1 |  |
| PSAP |  |
| ELOVL7 |  |
| GBA |  |

Table S18. Listing of baseline characteristics of individuals with rapid motor worsening

| ID | Gender | Age | Age at onset of PD | Disease duration (year) | Baseline L-dopa dose (mg) | Baseline LED (mg) | UPDRS III Score | | | |
| --- | --- | --- | --- | --- | --- | --- | --- | --- | --- | --- |
|  |  |  |  |  |  |  | baseline | Last observation | Annual change from baseline | Annual change from baseline (%) |
| 1 | Female | 85 | 71 | 15.0 | 600.0 | 798.0 | 33 | 65 | 15.8 | 48.0 |
| 2 | Female | 57 | 54 | 3.8 | 600.0 | 650.0 | 11 | 30 | 12.4 | 112.6 |
| 3 | Female | 75 | 66 | 10.0 | 1000.0 | 1200.0 | 24 | 48 | 9.4 | 39.3 |
| 4 | Female | 67 | 66 | 1.8 | 450.0 | 550.0 | 12 | 29 | 7.9 | 66.2 |
| 5 | Female | 70 | 66 | 5.0 | 300.0 | 550.0 | 17 | 35 | 7.3 | 42.9 |
| 6 | Male | 65 | 55 | 10.1 | 700.0 | 1181.0 | 2 | 19 | 6.8 | 341.5 |
| 7 | Male | 77 | 37 | 40.0 | 0.0 | 0.0 | 7 | 18 | 6.7 | 96.2 |
| 8 | Male | 79 | 73 | 7.0 | 0.0 | 200.0 | 12 | 28 | 6.3 | 52.7 |
| 9 | Male | 53 | 52 | 1.1 | 300.0 | 479.6 | 7 | 18 | 5.6 | 80.0 |
| 10 | Female | 78 | 66 | 12.4 | 800.0 | 1214.0 | 30 | 38 | 5.3 | 17.6 |
| 11 | Female | 68 | 66 | 1.1 | 300.0 | 300.0 | 11 | 19 | 5.1 | 46.8 |
| 12 | Male | 59 | 46 | 14.0 | 0.0 | 119.7 | 24 | 34 | 5.0 | 21.0 |
| 13 | Male | 75 | 74 | 1.9 | 450.0 | 550.0 | 18 | 30 | 5.0 | 27.9 |
| 14 | Male | 54 | 51 | 4.8 | 300.0 | 962.5 | 7 | 20 | 4.9 | 70.2 |
| 15 | Female | 70 | 61 | 10.0 | 600.0 | 898.0 | 9 | 21 | 4.7 | 52.7 |
| 16 | Female | 81 | 71 | 10.5 | 450.0 | 598.5 | 23 | 34 | 4.5 | 19.4 |
| 17 | Female | 83 | 82 | 1.3 | 0.0 | 100.0 | 13 | 19 | 4.1 | 31.2 |
| 18 | Male | 72 | 66 | 7.0 | 300.0 | 624.0 | 8 | 18 | 4.0 | 49.8 |
| 19 | Female | 61 | 59 | 1.4 | 0.0 | 100.0 | 22 | 27 | 3.9 | 17.7 |

Table S19. Comparison of baseline characteristics between patients with rapid motor worsening and those with stable or improved motor scores.

|  |  | Early progressors | | Patients with stable disease or improvement | | P value | Test method |
| --- | --- | --- | --- | --- | --- | --- | --- |
| Total number of patients |  | 19 |  | 57 |  |  |  |
| Gender | Male | 7 | (36.8%) | 24 | (42.1%) | *p=*0.791 | Fisher’s exact test |
|  | Female | 12 | (63.2%) | 33 | (57.9%) |  |  |
| Age | N | 19 |  | 57 |  | *p=*0.251 | Student's t-test |
|  | Mean | 68.5 |  | 65.7 |  |  |  |
|  | SD | 10.5 |  | 8.4 |  |  |  |
|  | Min | 46 |  | 48 |  |  |  |
|  | Median | 72.0 |  | 66.0 |  |  |  |
|  | Max | 85 |  | 83 |  |  |  |
|  | Unknown | 0 |  | 0 |  |  |  |
| Height (cm) | N | 15 |  | 57 |  | *p=*0.453 | Student's t-test |
|  | Mean | 159.1 |  | 161.3 |  |  |  |
|  | SD | 10.2 |  | 10.3 |  |  |  |
|  | Min | 141 |  | 143 |  |  |  |
|  | Median | 156.0 |  | 160.0 |  |  |  |
|  | Max | 179 |  | 180 |  |  |  |
|  | Unknown | 4 |  | 0 |  |  |  |
| Age at onset of PD | N | 19 |  | 57 |  | *p=*0.741 | Student's t-test |
|  | Mean | 61.5 |  | 60.6 |  |  |  |
|  | SD | 10.4 |  | 10.4 |  |  |  |
|  | Min | 38 |  | 28 |  |  |  |
|  | Median | 65.0 |  | 62.0 |  |  |  |
|  | Max | 76 |  | 76 |  |  |  |
|  | Unknown | 0 |  | 0 |  |  |  |
| Age at onset of PD | <50 | 3 | (15.8%) | 8 | (14.0%) | *p=*1.000 | Fisher’s exact test |
|  | ≧50 | 16 | (84.2%) | 49 | (86.0%) |  |  |
| Disease Duration (year) | N | 19 |  | 57 |  | *p=*0.119 | Student's t-test |
|  | Mean | 7.92 |  | 5.91 |  |  |  |
|  | SD | 4.00 |  | 5.01 |  |  |  |
|  | Min | 1.4 |  | 0.4 |  |  |  |
|  | Median | 8.60 |  | 4.00 |  |  |  |
|  | Max | 15.0 |  | 21.0 |  |  |  |
|  | Unknown | 0 |  | 0 |  |  |  |
| Disease duration | <Mean | 7 | (36.8%) | 37 | (64.9%) | *p=*0.059 | Fisher’s exact test |
|  | ≥Mean | 12 | (63.2%) | 20 | (35.1%) |  |  |
| Motor worsening surrogate ^a^ | N | 19 |  | 57 |  | *p=*0.072 | Student's t-test |
|  | Mean | 3.41 |  | 9.84 |  |  |  |
|  | SD | 3.62 |  | 15.14 |  |  |  |
|  | Min | 0.2 |  | 1.0 |  |  |  |
|  | Median | 2.14 |  | 5.17 |  |  |  |
|  | Max | 13.6 |  | 100.0 |  |  |  |
|  | Unknown | 0 |  | 0 |  |  |  |
| Family history of Parkinson’s disease | Yes | 3 | (15.8%) | 12 | (21.1%) | *p=*1.000 | Fisher’s exact test |
|  | No | 11 | (57.9%) | 37 | (64.9%) |  |  |
|  | Unknown | 5 | (26.3%) | 8 | (14.0%) |  |  |
| Time from the onset of motor symptoms to referral to specialized care (Juntendo University Hospital) | N | 19 |  | 57 |  | *p=*0.141 | Student's t-test |
|  | Mean | 2896.4 |  | 2227.7 |  |  |  |
|  | SD | 1449.1 |  | 1766.2 |  |  |  |
|  | Min | 687 |  | 309 |  |  |  |
|  | Median | 3251.0 |  | 1636.0 |  |  |  |
|  | Max | 5442 |  | 7620 |  |  |  |
|  | Unknown | 0 |  | 0 |  |  |  |
| Time from the onset of motor symptoms to referral to a specialized hospital (Juntendo University) | <Mean | 7 | (36.8%) | 36 | (63.2%) | *p=*0.062 | Fisher’s exact test |
|  | ≥Mean | 12 | (63.2%) | 21 | (36.8%) |  |  |

a. Motor worsening surrogate is defined as baseline UPDRS III score divided by disease duration.

1. Daida K, Funayama M, Li Y, et al. Identification of Disease-Associated Variants by Targeted Gene Panel Resequencing in Parkinson's Disease. *Front Neurol*. 2020;11:576465.
